# Sustainable Valorization of Grape Pomace Digestate as Fertilizer: Effects on the Agronomic and Biochemical Performance of *Calendula officinalis* L. under Variable Irrigation Regimes

**DOI:** 10.1021/acsomega.6c00987

**Published:** 2026-06-19

**Authors:** Luiz Eduardo Nochi Castro, Renata Bachin Mazzini-Guedes, Maycon Diego Ribeiro, Larissa Resende Matheus, Daiane Leticia Quirino de Souza, Leomara Floriano Ribeiro, Leda Maria Saragiotto Colpini, Tania Forster-Carneiro

**Affiliations:** † School of Food Engineering (FEA), State University of Campinas (UNICAMP), Campinas, SP 13083-862, Brazil; ‡ Advanced Campus of Jandaia do Sul, Federal University of Parana (UFPR), Jandaia do Sul, PR 86900-088, Brazil; § Graduate Program in Technology and Environmental Engineering, Palotina Sector, Federal University of Parana (UFPR), Palotina, PR 85953-128, Brazil; ∥ Institute of Chemistry (IQ), State University of Campinas (UNICAMP), Campinas, SP 13083-861, Brazil

## Abstract

This study explores the sustainable valorization of grape pomace digestate as a biofertilizer for the cultivation of *Calendula officinalis* L., a medicinal and edible flower with significant functional potential. Grape pomace, a major byproduct of winemaking, was subjected to anaerobic digestion to produce a nutrient-rich digestate applied at 0, 10, 20, and 30% concentrations under four irrigation regimes (57–232 mL day^–1^). Two seasonal greenhouse experiments evaluated agronomic performance, flower yield, and biochemical composition. Moderate to high digestate applications (20–30%) markedly enhanced flower yield and aerial biomass, while promoting the accumulation of proteins, lipids, soluble sugars, carotenoids, and phenolic compounds. Flowers grown under these conditions exhibited elevated β-carotene levels (80.97 mg 100 g^–1^) and strong antioxidant activity (>1300 μmol TEAC 100 g^–1^), supporting their potential in functional foods, nutraceuticals, and natural colorants. Seasonal variation influenced metabolic responses, with summer enhancing sugar and carotenoid synthesis and winter favoring pigment stability. The digestate was free from toxic elements and improved substrate fertility, confirming its agronomic safety and efficiency. A sustainability evaluation yielded an EcoScale score of 89.8, substantially higher than values reported in comparable literature, underscoring the high environmental performance and process greenness of this approach. Overall, this work presents a scalable, eco-efficient strategy to convert winery residues into high-value floral biomass, advancing circular bioeconomy principles and sustainable agricultural practices.

## Graphical abstract

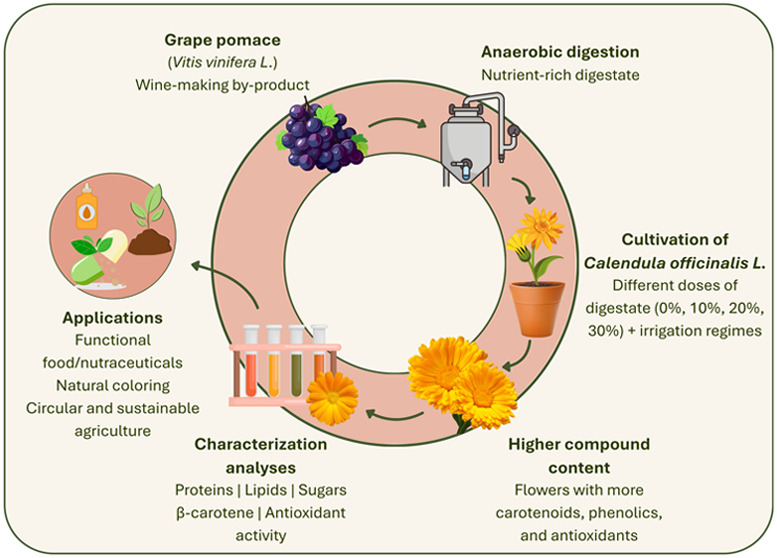

## 1 Introduction

The management of agro-industrial residues represents a major sustainability challenge for the food and beverage sector. Among these residues, grape pomace, the solid byproduct generated during grape processing for winemaking, accounts for roughly 20–25% of the total grape mass processed, generating an estimated 9–13 million tons annually worldwide.^1,2^ Composed mainly of skins, seeds, and stems, grape pomace is rich in organic matter, polyphenols, and essential plant nutrients. Traditionally discarded or used as low-value feed, this biomass has gained renewed attention as a resource for value-added applications within circular bioeconomy frameworks.^3^

Anaerobic digestion (AD) is one of the most promising valorization pathways for grape pomeace. The process converts organic matter into biogas and a nutrient-rich byproduct, the digestate, which retains most of the macronutrients (N, P, K) and micronutrients present in the original material. Reuse of digestate as an organic fertilizer can improve soil structure, enhance microbial activity, and reduce dependence on synthetic fertilizers.^4^ However, its agricultural use is still constrained by variable nutrient speciation, elevated salinity or electrical conductivity, ammonia toxicity, potential accumulation of heavy metals, and the presence of organic or microbiological contaminants.^5,6^ These factors can limit plant growth or soil health if they are not properly managed.^7^ While several studies have examined digestate application on staple crops such as cereals and forages, data remain scarce on its use for high-value horticultural and medicinal plants.^8,9^

*Calendula officinalis* L. (pot marigold) offers a suitable model for evaluating digestate as a sustainable fertilizer in specialty crop production. Although cultivated mainly for its ornamental and medicinal value rather than as a staple food crop, *C. officinalis* L. has broad agronomic and economic relevance.^10^ It is grown globally in small- to medium-scale systems for the extraction of bioactive compounds used in pharmaceuticals, cosmetics, and functional foods. Its flowers are rich in carotenoids, flavonoids, and triterpenoids with documented anti-inflammatory, antimicrobial, antioxidant, and wound healing activities.^11^ Moreover, as an edible flower increasingly marketed for nutraceutical and natural pigment applications, *C. officinalis* L. represents a promising niche crop for sustainable intensification strategies.^12^ Evaluating its response to organic amendments such as digestate could provide valuable insight into how such inputs influence biomass yield and phytochemical composition in nonstaple, high-value species.^13^

Despite the growing interest in digestate recycling, little is known about how grape pomace digestate specifically affects plant growth, nutrient uptake, and metabolite accumulation in medicinal or ornamental species.^14,15^ Existing research often lacks compositional fingerprinting of digestate, dose–response modeling, and assessments of interactive effects with water availability, factors critical to safe and efficient digestate utilization.^16^

This study addresses the existing knowledge gap by investigating, for the first time, the agronomic and biochemical responses of *C. officinalis* L. to graded applications of grape pomace digestate under varying irrigation regimes. It is hypothesized that moderate digestate doses enhance soil fertility and plant performance by supplying bioavailable nutrients and stimulating secondary metabolism, whereas excessive application may induce osmotic or ammonium stress. Through quantification of changes in substrate properties, flower yield, and phytochemical composition across two growing seasons, the study aims to (i) characterize the dose–response relationships of *C. officinalis* L. to digestate fertilization, (ii) identify threshold levels for optimal performance, and (iii) demonstrate a closed-loop valorization pathway that transforms winery residues into high-value edible biomass.

## 2 Materials and Methods

### 2.1 Materials

Seeds of *C. officinalis* L. cv. Pôr do Sol (Isla, Porto Alegre, RS, Brazil) were used for pot marigold sowing and cultivation. The substrate consisted of Carolina Soil (Carolina Soil, São José, SC, Brazil), which is based on Canadian peat, expanded vermiculite, expanded perlite, and roasted rice husks. Plastic sowing trays with 64 cells (each with a depth of 6 cm and a total volume of 29 cm^3^) and plastic pots with a capacity of 700 mL were used for cultivation. A caliper (Insize, São Paulo, SP, Brazil) was employed to measure flower diameter.

Acetone (C_3_H_6_O), aluminum chloride (AlCl_3_), ammonium molybdate ((NH_4_)_6_Mo_7_O_24_·4H_2_O), arsenic acid dibasic sodium salt (Na_2_HAsO_4_), bromocresol green (C_21_H_14_Br_4_O_5_S), catechin (C_15_H_14_O_6_), chloroform (CHCl_3_), citric acid (C_6_H_8_O_7_), copper sulfate (CuSO_4_), DPPH (2,2-diphenyl-1-picrylhydrazyl) (C_18_H_12_N_5_O_6_), ethanol (C_2_H_5_OH), ferric chloride (FeCl_3_), Folin-Ciocalteu reagent, gallic acid (C_7_H_6_O_5_), hydrochloric acid (HCl), sodium bicarbonate (NaHCO_3_), sodium hydroxide (NaOH), sodium nitrite (NaNO_2_), sodium sulfate (Na_2_SO_4_), sulfuric acid (H_2_SO_4_), methanol (CH_3_OH), methyl red (C_15_H_15_N_3_O_2_), potassium sodium tartrate (C_4_H_4_KNaO_6_·4H_2_O), potassium sulfate (K_2_SO_4_), selenium powder (Se), TPTZ (2,4,6-tris­(2-pyridyl)-s-triazine) (C_18_H_12_N_6_), and Trolox (C_14_H_18_O_4_) were purchased from Merck-Sigma (St. Louis, MO, United States). All chemicals were of analytical grade and used as received without further purification.

Grape (*Vitis vinifera*) pomace, used in AD to produce the digestate, was obtained from a local winery in Campinas, Brazil.

### 2.2 Obtaining Plant Material and Digestate

The digestate used in the experiments was obtained from a pilot-scale methanogenic reactor installed at the Laboratory of Bioengineering and Treatment of Water and Waste (BIOTAR) within the School of Food Engineering at the University of Campinas (Campinas, SP, Brazil). The 4.3 L stirred-tank reactor operated under semicontinuous conditions for 40 days at 36 °C, with mechanical stirring and pH maintained between 7.0 and 8.5 through NaOH addition. The reactor processed a substrate composed of grape pomace, water, and mesophilic inoculum sourced from an up flow anaerobic sludge blanket (UASB) reactor treating wastewater from a poultry slaughterhouse (Dacar Company, Tietê, SP, Brazil). The substrate composition consisted of 45% dried grape pomace (600 g; 1.16 L), 27.5% mesophilic inoculum (715 g; 0.71 L), and 22.5% water (0.71 L), occupying 60% of the total reactor volume (2.58 L), with 40% headspace (1.72 L). The reactor operated with a hydraulic retention time of 25.95 days, an organic loading rate of 9.90 g ODM L^–1^ d^–1^, and a volatile solids loading rate of 4.54 g TVS L^–1^ d^–1^. The mesophilic inoculum presented organic matter of 0.45 g O_2_ L^–1^, biogas composition of 68% CH_4_ during UASB operation, biomass retention time of 8 h, and pH of 7.3. At the end of the experiment, the digestate was separated and frozen at −18 °C until use as fertilizer. Additional operational conditions and reactor performance data are described in Castro et al. (2024).^8^

For the experiment, pot marigold (*C. officinalis* L.) was chosen for cultivation due to its production of edible flowers and its culinary and medicinal uses. The *Pôr do Sol* cultivar was selected for its good adaptation to different climates and its requirement of at least 4 h of full sunlight per day.

### 2.3 Substrate Characteristics and Fertilizer Composition

The substrate used was a commercial product (Carolina Soil, São José, SC, Brazil) composed of Canadian peat, expanded vermiculite, expanded perlite, and roasted rice hulls. Its composition is shown in Table S1 (Supporting Information).

The digestate was characterized through the evaluation of several physicochemical parameters, as shown in Table S2, including pH, electrical conductivity, ammonia nitrogen, alkalinity, total chemical oxygen demand (COD), total nitrogen, nitrate, nitrite, solids profile, phosphorus content, and soluble protein, following standard analytical protocols.^8,17,18^ Elemental composition was determined by inductively coupled plasma optical emission spectrometry (ICP-OES). In addition, volatile fatty acids (VFAs) were separated and quantified using high-performance liquid chromatography (HPLC) equipped with a refractive index detector (RID).^8^ Concentrations of propionic, isobutyric, and isovaleric acids were specifically measured.

### 2.4 Experimental Setup

The experiment was conducted in a 7 × 12 m nonacclimatized greenhouse, externally covered with a transparent polyethylene film, internally lined with a 50% black shading net, and with sides protected by a 20% white shading net. The greenhouse is located at 23°36′11″ S, 51°38′36″ W, at an altitude of 807 m. According to the Köppen classification, the regional climate is Cfa, characterized as a humid temperate climate with hot summers, with mean annual minimum and maximum temperatures of 17.4 and 27.2 °C, respectively. Two experimental cycles were conducted over the course of one year. The first began in March and lasted 124 days, while the second started in September and lasted 107 days. During the first cycle, the mean minimum and maximum air temperatures were 16.0 and 31.4 °C, with relative humidity ranging from 15.9 to 92.1%. In the second cycle, the mean minimum and maximum temperatures were 18.6 and 36.6 °C, with relative humidity ranging from 11.3 to 89.5%.

For each experiment, seeds of pot marigold “Pôr do Sol” were sown in 64-cell plastic trays. Germination occurred after 4 days, and seedlings were transplanted 20 days later into plastic pots with a capacity of 700 mL. Each pot was filled with 250 g of substrate containing digestate at the following treatment levels: 0% (control), 10, 20, and 30%. After a seven-day acclimation period, seedlings were placed on the experimental bench, where they were grown under four different irrigation regimes supplying 57, 118, 180, or 232 mL of water per day.

In both experimental cycles, 84 pots were prepared. However, to reduce edge effects, only 12 pots per treatment were evaluated, totaling 48 pots per cycle. The pots were arranged along irrigation lines organized by the digestate concentration and water volume treatment.

The irrigation system consisted of a 500 L reservoir, a 1/2 HP motor pump, a 120-mesh disc filter, ball valves, four solenoid valves, and an 8-station electronic controller. Water was delivered through 8 mm low-pressure drip microtubes with emitters spaced at 40 cm intervals. The system included one dedicated irrigation line for each water volume treatment as well as two additional border lines placed on either side of the bench. The entire system was operated automatically to deliver precise volumes according to the experimental design.

Treatments A, E, I, and M received a single daily irrigation at 06:00 AM, lasting 1 min and applying 57 mL per pot. Treatments B, F, J, and N were irrigated twice daily at 06:10 AM and 01:10 PM, each for 1 min, resulting in a total of 118 mL per pot. Treatments C, G, K, and O received three daily irrigations at 06:20 AM, 10:20 AM, and 02:20 PM, each for 1 min, totaling 180 mL per pot. Finally, treatments D, H, L, and P were irrigated four times daily at 06:30 AM, 09:30 AM, 12:30 PM, and 03:10 PM, each for 1 min, resulting in a total of 232 mL per pot.

### 2.5 Seed Germination, Fertilization Strategies, and Experimental Design

The experiment was designed in a 4 × 4 factorial arrangement, combining four irrigation volumes (57, 118, 180, and 232 mL) with four digestate contents (0, 10, 20, and 30%), resulting in a total of 16 experimental conditions, as shown in Table 1. Each condition was conducted in quadruplicate to ensure reproducibility. Samples were prepared according to predefined levels for each factor and subjected to the corresponding analyses, allowing for the evaluation of the effects of irrigation volume and digestate content on the measured responses.

**Table 1:** Experiment Design

Code	Test^a^	Watering rate (mL)	Digestate content (%)
A	L1T0	57	0
B	L2T0	118	0
C	L3T0	180	0
D	L4T0	232	0
E	L1T1	57	10
F	L2T1	118	10
G	L3T1	180	10
H	L4T1	232	10
I	L1T2	57	20
J	L2T2	118	20
K	L3T2	180	20
L	L4T2	232	20
M	L1T3	57	30
N	L2T3	118	30
O	L3T3	180	30
P	L4T3	232	30

a L represents the watering rate, and T represents the digestate contents, with the numbers 1 to 4 indicating the levels used for each parameter.

### 2.6 Agronomic Characteristics of the Plants

To assess the effect of digestate content and irrigation volume on growth, flowering, and quality parameters of pot marigold plants, several measurements were performed. These included the number of flowers, flower diameter (measured with a caliper, INSIZE, model 1130–150A, São Paulo, SP, Brazil, at its maximum opening), flowering time (from the appearance of the first flower bud to the harvest of the last flower), harvest period (from the first to the last flower harvest), plant life cycle (from germination to plant senescence), plant height (measured with a ruler from the substrate level to the highest point of the plant), leaf area (using a leaf area meter, TECNAL, model CI-202, Piracicaba, SP, Brazil), fresh mass of the aerial part (using a precision scale, BEL Engineering, model M5, Monza, MB, Italy), and dry mass of the aerial part and roots (using the same precision scale, after drying in a forced-air circulation oven, TE-394/3, TECNAL, Piracicaba, SP, Brazil, at 65 °C until constant weight).

### 2.7 Pot Marigold Flower Characterization

Pot marigold flowers (*C. officinalis*) were harvested, freeze-dried using a lyophilizer (LIOBRAS, model Liotop LP820, São Carlos, Brazil), and ground with a ball mill (SOLAB, model SL-38, São Paulo, Brazil). The lyophilized flowers (PMF) were analyzed using various techniques.

Dry matter and ash content were determined gravimetrically: the flowers were dried in a convection oven (SolidSteel, model SSDcr-110L, Piracicaba, Brazil) at 105 °C until a constant weight was reached, then calcinated at 550 °C in a muffle furnace (Lucadema, model LUCA2000G, São José do Rio Preto, Brazil), following ASTM E870 standards (ASTM E870–82, 2019).

Reducing, nonreducing, and total sugars were measured using the Somogyi–Nelson method.^19^ Lipid composition was analyzed through cold extraction with a chloroform/methanol mixture.^20^ Nitrogen and crude protein contents were determined by the Kjeldahl method.

Chlorophyll a and b, total chlorophyll, total carotenoid content, and β-carotene were determined using a modified methodology adapted from Oliveira,^21^ employing 80% acetone as the extraction solvent.

To analyze the bioactive compounds present in PMF, an extract was prepared using ethanol and water. A 1:50 (w v^–1^) ratio of PMF to extracting solvent (40:60 ethanol:water, v^–1^ v^–1^) was used, following a methodology from Castro and collaborators.^22^

The determination of total phenolic compounds was carried out using a modified procedure based on the method of Swain and Hillis.^23^ Total flavonoid content was measured following the approach proposed by Meyers and colleagues.^24^ The antioxidant potential was assessed through two analytical techniques: the DPPH (1,1-diphenyl-2-picrylhydrazyl) radical scavenging assay and the ferric reducing antioxidant power (FRAP) assay, according to the protocol described by Benzie and Strain.^25^

The color characteristics of the extracts were analyzed using the CIELab color system. The coordinates L* (lightness), a* (red–green axis), and b* (blue–yellow axis) were obtained from transmittance readings recorded every 5 nm across the 340–830 nm range with a UV–vis spectrophotometer (Bell Photonics, model UV-M51, Piracicaba, Brazil).^26^ Chroma (C*) and hue angle (H°) were subsequently derived following the procedure described by Castro and collaborators.^27^

#### 2.7.1 Volatile Fatty Acids Profile (VFAs)

For fatty acid methyl ester analysis, 10 mg of flower oil was accurately weighed using an analytical balance with four decimal places (AUY220, Shimadzu, Kyoto, Japan) and solubilized in 500 μL of hexane PA (Hexano PA, Synth, Diadema, SP, Brazil). Then, 500 μL of sodium methoxide solution (2 mol L^–1^, Sigma-Aldrich, St. Louis, MO, USA) was added, and the mixture was homogenized. The reaction mixture was stirred at 200 rpm for 5 min using a digital orbital shaker (MA140/CFT, Marconi, Piracicaba, SP, Brazil).

Following the reaction, 80 mg of sodium chloride (NaCl, Dinâmica, Indaiatuba, SP, Brazil) and 100 mg of anhydrous sodium sulfate (Na_2_SO_4_, Vetec, Rio de Janeiro, RJ, Brazil) were added to assist phase separation and drying. The tubes were vortexed for 1 min (Vortex MA162, Marconi, Piracicaba, SP, Brazil), then centrifuged at 4000 rpm for 2 min (Centrifuge 80–2B, Centribio, São Paulo, SP, Brazil). The supernatant was collected and filtered using 13 mm nylon syringe filters with 0.45 μm pore size (Analytica, São Paulo, SP, Brazil) and transferred into chromatographic vials (Agilent, Santa Clara, CA, USA) for analysis.

Gas chromatography coupled with flame ionization detection (GC-FID) was carried out on a Shimadzu GC-2010 Plus system (Kyoto, Japan), fitted with a Nukol capillary column (30 m × 0.25 mm i.d., 0.25 μm film thickness; Supelco, Bellefonte, PA, USA). Samples were introduced in split mode (1:50). The injector operated at 220 °C under a pressure of 143.4 kPa, with a linear carrier gas velocity of 40.3 cm s^–1^. The total flow rate was 87.6 mL min^–1^, comprising a column flow of 1.62 mL min^–1^ and a purge flow of 5.0 mL min^–1^. Nitrogen (White Martins, São Paulo, Brazil) served as both carrier and purge gas.

The oven program initiated with an isothermal step at 120 °C for 1 min, followed by a temperature increase of 1.5 °C min^–1^ until reaching 218 °C, where it was maintained for 10 min. Quantification of compounds was achieved using a calibration curve prepared from the Supelco 37 Component FAME Mix (Supelco, Bellefonte, PA, USA).

### 2.8 Ecological Greenness Assessment

The EcoScale assessment was applied to evaluate the environmental sustainability of the extraction method used for determining the total carotenoid content in PMF. The analysis incorporated multiple criteria, including safety, choice of solvents, economic feasibility and availability, operational setup, temperature requirements, and procedural aspects. For comparison, EcoScale scores reported in related studies were also considered.^28^

To standardize the evaluation, the parameters were normalized according to the total carotenoid yield, with the highest yield obtained experimentally defined as 100%. Final scores ranged from 1 to 100, where 1 reflects the least sustainable process and 100 denotes the most environmentally friendly alternatives.

### 2.9 Statistical Analysis

All results are expressed as mean values with standard deviations from three independent replicates. Statistical evaluation was carried out using Statistica software (StatSoft Inc., Tulsa, USA). Analysis of variance (ANOVA) followed by Tukey’s post hoc test (*p* ≤ 0.05) was applied to determine significant differences between group means. In addition, principal component analysis (PCA) was employed to examine data structure, reduce dimensionality, and highlight the variables most responsible for variation among treatments.

## 3 Results and Discussion

### 3.1 Grape Pomace Digestate Characterization

After 40 days of operation, the digestate in the AD reactor exhibited low levels of total solids and COD. The performance of anaerobic systems is strongly dependent on the availability of organic substrates, which serve as the primary energy source for the microorganisms driving the digestion process.^29^ As the organic load within the reactor declined, process efficiency and yield decreased, which could negatively affect biogas generation.^30^ At this stage, the operational alternatives were either to increase the feedstock solid concentration or to end the trial. Since the main performance targets, such as the amount of grape pomace supplied, biogas output, methane recovery, and associated energy production (electricity and heat), had already been achieved, the experiment was concluded after 40 days. The principal physicochemical properties of the digestate employed for PMF cultivation are summarized in Table S2.

The pH of the digestate after AD was approximately 8.52, which indicates that the reactor maintained an optimal pH level for methane production.^30^ The electrical conductivity was measured at 0.24 dS m^–1^. Typically, the electrical conductivity of digestate should not exceed 40 dS m^–1^. However, no such effects were observed in the digestate used in this experiment.^31^

After 40 days of AD, the alkalinity of the digestate was around 0.4 g CaCO_3_ g^–1^, which is within the ideal range for AD.^32^ Alkalinity values between 0.1 and 0.5 g CaCO_3_ g^–1^ are highly beneficial for methane production, as they support the activity of the methanogenic microbial consortium.^33^

Ammonia in digestate is present in two forms: free ammonia (NH_3_) and ammonium ion (NH_4_^+^), both generated during the anaerobic breakdown of proteins, urea, and nucleic acids.^34^ The measured ammonia nitrogen content of the digestate was around 4.87 mg NH_3_ 100 g^–1^. This concentration is adequate to sustain microbial activity and methane generation, since values exceeding 100 mg NH_3_ 100 g^–1^ may hinder the AD process.^35^ In addition, this level of ammonia is expected to be favorable for the growth of pot marigold.^36^

The digestate obtained from grape pomace, with a total nitrogen content of approximately 1.65 g 100 g^–1^, presents potential as a nutrient-rich amendment for soil, particularly for cultivating crops like pot marigold.^37^ The total nitrogen present in the digestate is beneficial for plant growth, as nitrogen is an essential nutrient for vegetative growth and flowering. The moderate nitrogen levels in the digestate may contribute to enhancing the overall health and development of pot marigold plants, which are known for their sensitivity to nutrient imbalances. Additionally, the low levels of nitrate (NO_3_^–^) and nitrite (NO_2_^–^) in the digestate (<0.010 g 100 g^–1^) make it a safer alternative for use in agricultural applications, particularly in organic farming.^38^ Excessive nitrates and nitrites can lead to nutrient leaching, potentially contaminating water sources and posing risks to human and animal health. The low presence of these compounds in the digestate suggests that its application in pot marigold cultivation is less likely to contribute to environmental pollution, ensuring a more sustainable farming practice.^39^

The presence of phosphorus in the digestate can be crucial for plant growth, particularly in supporting root development, energy transfer, and flower formation. Although the phosphorus concentration in the digestate is modest (∼5 mg 100 g^–1^), its presence could still support pot marigold growth, especially when combined with other nutrients present in the digestate, such as potassium, nitrogen, and carbon.^40^

The solid fraction of the grape pomace digestate offers several benefits for pot marigold cultivation. The inorganic minerals in the fixed solids will support the plant’s nutrient needs, especially for growth and flowering.^41^ The organic matter in the volatile solids will contribute to nutrient cycling and enhance soil quality by improving soil structure, microbial activity, and water retention.^42^ A similar process occurs in substrates used for plant cultivation.

Given the relatively high organic content (3.85 g TVS 100 g^–1^), the digestate could be an effective soil amendment for pot marigold, particularly in soils that may be deficient in organic matter. The gradual decomposition of volatile solids would provide a slow-release source of nutrients, reduce the risk of nutrient leaching, and make the fertilization process more efficient.^43^ Additionally, the digestate’s ability to improve soil structure can help pot marigold plants thrive, as they benefit from well-aerated, nutrient-rich soil that retains moisture while allowing proper root respiration.^44^

Additionally, the solid portion of the digestate offers environmental advantages, functioning as a sustainable substitute for synthetic fertilizers.^45^ Utilizing organic residues such as grape pomace as digestate not only promotes waste recycling but also mitigates the environmental issues linked to conventional fertilizers, including nutrient leaching and soil degradation.^46^

The digestate from grape pomace contains 5.41 g 100 g^–1^ soluble protein, a valuable component for enhancing soil fertility and supporting plant growth.^47^ Soluble proteins in the digestate are primarily composed of amino acids, peptides, and other nitrogen-containing compounds that are readily available for plant uptake.^48^ These proteins are essential for plant growth, as they play key roles in processes such as cell division, enzyme activity, and overall metabolism.^49^ The amino acids and nitrogen-rich compounds provide a direct source of nutrition, promoting healthy plant development and improving flowering.^41^ Pot marigold, like many plants, requires an adequate supply of nitrogen for optimal growth, and the soluble proteins in the digestate can contribute to this requirement in a bioavailable form.^44^ The release of these compounds into the soil through the slow decomposition of the digestate will ensure a steady supply of nitrogen, reducing the need for synthetic fertilizers and minimizing the risk of nutrient imbalances.^46^

Chemical oxygen demand is a crucial indicator for assessing the performance of AD processes.^50^ During fermentation, methanogenic microorganisms oxidize organic compounds in an oxygen-free environment, maintaining a redox balance. A marked reduction in COD typically indicates effective degradation of organic matter and is closely linked to methane production.^51^ COD is also converted into soluble compounds and VFAs, which provide substrates for methanogenic microorganisms, supporting ongoing methane generation.^52^ In the present study, the digestate showed relatively low total solids (∼5.67 g 100 g^–1^) and COD (∼4.69 g O_2_ g^–1^), suggesting that a large portion of the organic material was decomposed during AD.^8^ The performance of anaerobic reactors is strongly influenced by the availability of organic substrates to sustain microbial activity. Consequently, the reduced COD levels reflect efficient organic matter conversion, enhancing resource recovery and reducing the environmental impact when the digestate is applied as a soil amendment or fertilizer.^53^

The carbon-to-nitrogen (C/N) ratio of the digestate obtained from grape pomace was measured to be 17.79. The C/N ratio reflects the balance between carbon and nitrogen in the digestate, influencing its nutrient release dynamics and the decomposition rate in the soil. A C/N ratio of 17.79 is considered relatively balanced, as a typical C/N ratio for composted organic materials falls within a range of 15–30.^8^ This ratio indicates that the digestate contains enough carbon to support microbial activity, which is essential for the decomposition process, while also providing an adequate nitrogen supply for plant growth.^54^ The presence of both carbon and nitrogen in suitable proportions enables the digestate to be slowly broken down by soil microbes, releasing nutrients over time in a manner that is beneficial for plant growth.^46^

The digestate obtained from the AD of grape pomace contains notable concentrations of VFAs, with propionic, isobutyric, and isovaleric acids being the predominant types. These VFAs are intermediate products formed during the breakdown of complex organic matter under anaerobic conditions.^55^ Their presence in the digestate is significant for both the digestion process and the potential agricultural applications of the final product. From a process perspective, the accumulation of these VFAs indicates active microbial metabolism, particularly the fermentative activity of acidogenic and acetogenic bacteria.^56^ Propionic acid is a common byproduct of carbohydrate fermentation, while isobutyric and isovaleric acids often result from the breakdown of amino acids such as valine and leucine.^57^ The presence of these branched-chain VFAs suggests effective degradation of the protein-rich and fibrous components of grape pomace, reflecting the diverse microbial activity within the anaerobic reactor.^8^

VFAs contribute to the gradual release of nutrients, supporting sustained plant development and reducing the immediate need for additional chemical fertilizers.^45^ Additionally, VFAs, such as propionic acid, possess natural antimicrobial properties that can help suppress certain soil-borne pathogens, thereby contributing to a healthier rhizosphere environment. However, it is essential to manage VFA concentrations carefully, as excessive levels can lead to phytotoxicity or soil acidification.^43^ In the case of the grape pomace digestate studied here, the balanced presence of VFAs (∼5.7 g VFAs 100 g^–1^ grape pomace) complements its overall profile as a promising organic amendment, enhancing its suitability for promoting vigorous growth and flowering in pot marigold cultivation.

Finally, the elemental composition of the digestate was predominantly composed of carbon (C), sodium (Na), and nitrogen (N), followed by notable amounts of iron (Fe), aluminum (Al), and phosphorus (P). Boron (B), potassium (K), magnesium (Mg), and selenium (Se) were also present, completing the nutrient profile of the digestate. Notably, potentially harmful elements such as arsenic (As), mercury (Hg), and lead (Pb) were absent, confirming the digestate’s safety for agricultural applications. Additionally, the presence of key macronutrients—nitrogen, phosphorus, and potassium (NPK)—underscores its value as an organic fertilizer.^58^ This nutrient-rich profile supports the cultivation of various crops, including ornamental, medicinal, and edible plants like pot marigold, thereby promoting sustainable and environmentally friendly farming practices.^8^

### 3.2 Agronomic Parameters

The results of the agronomic parameters for pot marigold cultivation are presented in Tables 2 and 3, for experiments 1 and 2, respectively. Statistical analysis was performed using Tukey’s test (*p* ≤ 0.05) to compare differences among treatments. Treatments that were not statistically different were grouped, while those with significant differences were placed in separate groups according to the test outcomes.

**Table 2:** Agronomic Parameters of Pot Marigold Cultivation Experiment 1

	**Agronomic parameters** ^a^									
**Sample**	**Number of flowers (dimensionless)**	**Flower diameter**(mm)	**Harvest time (days)**	**Flowering time (days)**	**Plant life cycle (days)**	**Plant height (cm)**	**Leaf area**(cm^**2**^ **)**	**Fresh matter of the aerial part (g)**	**Dry matter of the aerial part (g)**	**Root dry matter (g)**
**A**	13 ± 1^ab^	41.02 ± 1.82^a^	35 ± 2^a^	84 ± 2^ab^	118 ± 2^a^	15.40 ± 1.67^a^	122.4 ± 25.07^a^	7.52 ± 2.15^a^	4.24 ± 0.47^a^	4.53 ± 0.46^a^
**B**	13 ± 1^ab^	49.01 ± 1.49^ab^	29 ± 2^a^	78 ± 5^ab^	107 ± 6^a^	17.87 ± 2.89^a^	238.4 ± 30.01^abcd^	20.06 ± 1.54^abc^	5.17 ± 0.37^a^	7.19 ± 1.41^ab^
**C**	15 ± 3^b^	50.31 ± 2.33^ab^	30 ± 1^a^	82 ± 9^ab^	112 ± 9^a^	18.70 ± 2.53^a^	256.5 ± 32.23^abcd^	24.87 ± 0.93^bcd^	5.70 ± 0.46^a^	12.64 ± 2.62^ab^
**D**	12 ± 1^ab^	51.95 ± 1.83^b^	29 ± 2^a^	86 ± 6^ab^	114 ± 5^a^	17.40 ± 0.67^a^	300.3 ± 39.66^bcd^	23.78 ± 4.55^abcd^	5.54 ± 0.98^a^	6.14 ± 1.21^ab^
**E**	12 ± 1^ab^	48.20 ± 0.33^ab^	36 ± 2^a^	77 ± 5^ab^	113 ± 4^a^	15.83 ± 1.11^a^	198.8 ± 56.04^abc^	13.24 ± 7.00^abc^	4.46 ± 0.85^a^	4.42 ± 1.57^a^
**F**	12 ± 0^ab^	54.04 ± 1.95^b^	34 ± 4^a^	78 ± 4^ab^	112 ± 7^a^	16.77 ± 1.64^a^	295.2 ± 41.78^bcd^	22.70 ± 3.57^abcd^	6.01 ± 0.69^a^	8.40 ± 4.54^ab^
**G**	12 ± 3^ab^	53.27 ± 2.75^b^	37 ± 2^a^	85 ± 2^ab^	123 ± 1^a^	13.87 ± 1.38^a^	400.5 ± 45.60^d^	37.74 ± 9.77^d^	5.74 ± 0.56^a^	13.11 ± 0.80^ab^
**H**	16 ± 4^b^	50.28 ± 1.12^ab^	29 ± 0^a^	93 ± 3^b^	122 ± 3^a^	15.40 ± 0.67^a^	289.4 ± 26.30^abcd^	25.79 ± 3.65^bcd^	4.86 ± 0.79^a^	8.87 ± 2.55^ab^
**I**	16 ± 4^b^	40.46 ± 2.59^a^	34 ± 6^a^	78 ± 11^ab^	112 ± 8^a^	14.97 ± 1.16^a^	139.9 ± 40.86^ab^	10.73 ± 4.56^ab^	3.88 ± 0.25^a^	4.89 ± 0.82^ab^
**J**	13 ± 1^ab^	48.96 ± 3.30^ab^	32 ± 7^a^	82 ± 3^ab^	114 ± 8^a^	16.70 ± 2.40^a^	283.6 ± 34.37^abcd^	25.11 ± 2.95^bcd^	5.14 ± 0.25^a^	16.99 ± 7.70^b^
**K**	13 ± 2^ab^	55.53 ± 2.71^b^	34 ± 2^a^	79 ± 9^ab^	113 ± 9^a^	17.00 ± 1.20^a^	346.7 ± 82.57^cd^	27.53 ± 4.82^cd^	5.06 ± 0.98^a^	11.66 ± 5.76^ab^
**L**	14 ± 2^b^	50.38 ± 2.48^ab^	29 ± 2^a^	86 ± 6^ab^	115 ± 5^a^	16.17 ± 1.49^a^	275.2 ± 52.07^abcd^	22.00 ± 2.35^abcd^	4.87 ± 0.53^a^	3.80 ± 1.39^a^
**M**	12 ± 1^ab^	47.56 ± 1.22^ab^	38 ± 3^a^	77 ± 11^ab^	115 ± 8^a^	16.10 ± 0.87^a^	228.2 ± 43.55^abc^	16.75 ± 2.08^abc^	3.80 ± 0.26^a^	4.44 ± 0.09^a^
**N**	18 ± 4^b^	47.72 ± 3.43^ab^	36 ± 2^a^	81 ± 10^ab^	117 ± 9^a^	18.47 ± 1.89^a^	300.0 ± 13.67^bcd^	27.18 ± 1.28^bcd^	5.19 ± 0.44^a^	8.96 ± 3.41^ab^
**O**	14 ± 2^b^	52.89 ± 3.10^b^	35 ± 4^a^	72 ± 10^ab^	107 ± 6^a^	17.10 ± 0.67^a^	318.5 ± 19.74^cd^	27.07 ± 3.11^bcd^	5.43 ± 0.65^a^	4.87 ± 2.32^ab^
**P**	4 ± 1^a^	56.67 ± 4.44^b^	39 ± 4^a^	60 ± 4^a^	99 ± 0^a^	16.67 ± 2.11^a^	311.8 ± 30.68^cd^	3.58 ± 1.19^abcd^	3.58 ± 0.44^a^	2.18 ± 0.37^a^

a Results expressed as the mean ± standard deviation of triplicates. Different lowercase letters indicate significant differences among treatments based on Tukey’s test at *p* ≤ 0.05. Sample codes: A: 57 mL water and 0% digestate; B: 118 mL water and 0% digestate; C: 180 mL water and 0% digestate; D: 232 mL water and 0% digestate; E: 57 mL water and 10% digestate; F: 118 mL water and 10% digestate; G: 180 mL water and 10% digestate; H: 232 mL water and 10% digestate; I: 57 mL water and 20% digestate; J: 118 mL water and 20% digestate; K: 180 mL water and 20% digestate; L: 232 mL water and 20% digestate; M: 57 mL water and 30% digestate; N: 118 mL water and 30% digestate; O: 180 mL water and 30% digestate; P: 232 mL water and 30% digestate.

**Table 3:** Agronomic Parameters of Pot Marigold Cultivation Experiment 2

	**Agronomic parameters** ^a^									
**Sample**	**Number of flowers (dimensionless)**	**Flower diameter**(mm)	**Harvest time (days)**	**Flowering time (days)**	**Plant life cycle (days)**	**Plant height**(cm)	**Leaf area**(cm^**2**^ **)**	**Fresh matter of the aerial part (g)**	**Dry matter of the aerial part (g)**	**Root dry matter (g)**
**A**	9 ± 1^b^	41.87 ± 1.72^a^	41 ± 3^a^	64 ± 3^c^	105 ± 0^a^	15.50 ± 0.73^abcd^	294.6 ± 41.40^ab^	15.47 ± 0.61^abc^	3.11 ± 0.02^ab^	4.36 ± 2.02^a^
**B**	4 ± 0^ab^	49.54 ± 2.32^a^	45 ± 6^ab^	57 ± 10^bc^	101 ± 5^a^	13.57 ± 0.09^abc^	351.5 ± 5.44^ab^	14.76 ± 0.75^abc^	2.98 ± 0.24^ab^	4.87 ± 0.66^ab^
**C**	8 ± 1^ab^	49.28 ± 0.78^a^	37 ± 0^a^	65 ± 5^c^	102 ± 5^a^	15.40 ± 1.73^abcd^	320.6 ± 45.09^ab^	17.79 ± 0.98^abc^	3.67 ± 0.10^b^	10.59 ± 1.84^b^
**D**	6 ± 1^ab^	49.60 ± 2.43^a^	36 ± 0^a^	69 ± 0^c^	105 ± 0^a^	15.70 ± 0.67^abcd^	293.1 ± 8.21^ab^	18.69 ± 0.18^abc^	3.56 ± 0.04^ab^	6.18 ± 0.20^ab^
**E**	7 ± 2^ab^	42.38 ± 1.96^a^	46 ± 1^ab^	55 ± 4^abc^	102 ± 5^a^	14.30 ± 0.27^abcd^	277.3 ± 51.20^ab^	13.31 ± 2.09^abc^	2.80 ± 0.05^ab^	5.08 ± 1.02^ab^
**F**	8 ± 3^ab^	44.97 ± 2.98^a^	41 ± 4^a^	63 ± 10^c^	105 ± 7^a^	17.93 ± 1.31^d^	318.5 ± 68.39^ab^	18.19 ± 1.70^abc^	3.61 ± 0.53^b^	7.11 ± 1.37^ab^
**G**	6 ± 1^ab^	51.44 ± 1.44^a^	38 ± 3^a^	47 ± 7^abc^	85 ± 6^a^	16.27 ± 1.98^bcd^	420.4 ± 18.45^b^	21.19 ± 1.52^bc^	3.75 ± 0.34^b^	6.15 ± 1.23^ab^
**H**	6 ± 1^ab^	50.41 ± 2.36^a^	39 ± 3^a^	51 ± 4^abc^	90 ± 6^a^	14.53 ± 0.44^abcd^	320.6 ± 47.98^ab^	17.97 ± 1.64^abc^	3.11 ± 0.26^ab^	4.45 ± 0.86^a^
**I**	8 ± 2^ab^	42.34 ± 1.19^a^	42 ± 3^ab^	58 ± 13^bc^	100 ± 12^a^	15.13 ± 0.76^abcd^	233.1 ± 37.56^ab^	11.42 ± 3.78^ab^	2.79 ± 0.49^ab^	4.19 ± 0.13^a^
**J**	4 ± 2^ab^	53.41 ± 1.99^a^	49 ± 1^abc^	40 ± 10^abc^	89 ± 11^a^	14.83 ± 0.51^abcd^	342.9 ± 78.89^ab^	16.62 ± 2.24^abc^	2.67 ± 0.36^ab^	5.74 ± 1.17^ab^
**K**	9 ± 1^b^	50.59 ± 1.70^a^	36 ± 0^a^	66 ± 5^c^	102 ± 5^a^	16.90 ± 1.33^cd^	322.6 ± 53.93^ab^	17.90 ± 0.22^abc^	3.45 ± 0.26^ab^	5.94 ± 1.62^ab^
**L**	7 ± 2^ab^	50.86 ± 2.85^a^	45 ± 3^ab^	64 ± 8^c^	110 ± 6^a^	15.13 ± 0.98^abcd^	304.4 ± 16.36^ab^	21.93 ± 5.62^c^	3.57 ± 0.26^b^	8.05 ± 2.45^ab^
**M**	8 ± 4^ab^	43.83 ± 2.48^a^	56 ± 4^bc^	42 ± 20^abc^	98 ± 18^a^	14.00 ± 0.20^abcd^	208.2 ± 47.29^a^	10.17 ± 4.65^a^	2.56 ± 0.43^ab^	5.85 ± 1.97^ab^
**N**	4 ± 1^ab^	49.14 ± 6.16^a^	62 ± 4^c^	19 ± 4^a^	81 ± 0^a^	12.30 ± 0.20^ab^	376.2 ± 37.29^ab^	17.14 ± 1.36^abc^	2.36 ± 0.26^a^	4.03 ± 0.05^a^
**O**	6 ± 2^ab^	51.98 ± 3.13^a^	50 ± 5^abc^	48 ± 10^abc^	98 ± 5^a^	14.87 ± 1.22^abcd^	413.6 ± 87.84^ab^	21.00 ± 2.24^bc^	3.67 ± 0.03^b^	5.54 ± 0.68^ab^
**P**	2 ± 0^a^	47.83 ± 5.28^a^	63 ± 8^c^	22 ± 9^ab^	85 ± 6^a^	11.70 ± 0.73^a^	415.6 ± 83.38^b^	18.30 ± 2.50^abc^	2.59 ± 0.25^ab^	6.82 ± 2.15^ab^

a Results expressed as the mean ± standard deviation of triplicates. Different lowercase letters indicate significant differences among treatments based on Tukey’s test at *p* ≤ 0.05. Sample codes: A: 57 mL water and 0% digestate; B: 118 mL water and 0% digestate; C: 180 mL water and 0% digestate; D: 232 mL water and 0% digestate; E: 57 mL water and 10% digestate; F: 118 mL water and 10% digestate; G: 180 mL water and 10% digestate; H: 232 mL water and 10% digestate; I: 57 mL water and 20% digestate; J: 118 mL water and 20% digestate; K: 180 mL water and 20% digestate; L: 232 mL water and 20% digestate; M: 57 mL water and 30% digestate; N: 118 mL water and 30% digestate; O: 180 mL water and 30% digestate; P: 232 mL water and 30% digestate.

In experiment 1, the number of flowers was notably higher in the treatments with moderate to high digestate concentrations combined with varying watering rates. Specifically, treatments H (232 mL of water +10% digestate), I (57 mL of watering rate +20% digestate), and N (118 mL of watering rate +30% digestate) produced between 16 and 18 flowers. These treatments formed a distinct group in the Tukey test, demonstrating superior performance over the other treatments. Conversely, treatment P (232 mL of watering rate +30% digestate), which used the highest combination of both digestate concentration and water volume, resulted in the lowest flower production with only four flowers. This significant reduction likely reflects an adverse effect of excess nutrients and water, potentially leading to nutrient imbalances or phytotoxicity, which in turn impairs floral development.^59^

Regarding flower diameter, treatment P produced the largest flowers, reaching an average diameter of 56.67 mm. Another treatment, K (180 mL of watering rate +20% digestate), followed closely at 55.53 mm. These two treatments formed a distinct group in Tukey’s test, indicating their advantage in promoting larger flower size. In contrast, treatments with lower digestate concentrations and watering rates, such as A (57 mL of watering rate +0% digestate) and I, consistently produced smaller flowers, underscoring the importance of adequate nutrient and water supply for optimal flower development.^60^

Harvest time did not show significant variation among treatments. According to Tukey’s test, all treatments were grouped together, indicating that the different combinations of digestate and watering rate did not influence the timing of harvest.

In terms of flowering time, treatment P again stood out, achieving flowering in just 60 days and forming a distinct group in Tukey’s test. This suggests that higher nutrient availability may accelerate the transition to the flowering stage, whereas treatments with lower nutrient levels, such as H, experienced delayed flowering.^61^

The duration of the plant life cycle did not differ significantly across treatments, as all combinations of digestate and watering rates were grouped together, indicating that these factors did not significantly affect the overall life cycle of the pot marigold plants.

Plant height was similarly unaffected, with no significant differences observed among treatments. This suggests that variations in water and digestate supply, besides other external factors, do not play a crucial role in determining the vertical growth in pot marigolds. As it also happens with other plant species, their leaves grow as a rosette, which is a strong genetic characteristic controlling such a habit.^62^

The leaf area, however, was positively influenced by certain treatments. Treatment G (180 mL of watering rate +10% digestate) resulted in the largest leaf area (400.5 cm^2^), forming a distinct group in Tukey analysis. Other treatments with higher nutrient and watering rates, such as K, O (180 mL of watering rate +30% digestate), and P, also performed well. Meanwhile, treatments with no digestate and/or low water availability, such as A and I, consistently produced smaller leaf areas. This highlights the benefits of balanced water rates and nutrient inputs for vegetative growth.

The fresh matter of the aerial parts followed a similar pattern. Treatment G, which also maximized the leaf area, yielded the highest fresh matter (37.74 g), indicating that these conditions promote overall plant vigor. In contrast, treatment P, despite encouraging early flowering and larger flowers, resulted in the lowest fresh matter (3.58 g), suggesting that excessive nutrients and water might prioritize reproductive growth at the expense of vegetative biomass accumulation.^63^

The dry matter of the aerial part did not show significant differences among treatments, indicating that while fresh matter and leaf area were affected, the accumulation of dry matter remained relatively stable regardless of water and digestate levels.

Root dry matter was highest in treatment J (118 mL of watering rate +20% digestate) with 16.99 g, forming a distinct group in the Tukey analysis. This suggests that moderate nutrient and water availability are optimal for root development. Treatments A and P had the lowest root dry matter, showing that both digestate and water low inputs and excessive levels were less favorable, inhibiting root growth.

In summary, treatment P was the most effective in producing larger flowers and accelerating flowering; however, it resulted in the lowest number of flowers, which may be a drawback for producers seeking a high flower yield. Treatment N stood out by producing the highest number of flowers, making it the best option when the quantity is prioritized. Meanwhile, treatment G was most effective in increasing the leaf area and fresh mass of the aerial part, and treatment J promoted the greatest root development.

In experiment 2, the number of flowers was notably higher in treatments A and K, both of which produced 9 flowers. These treatments formed a distinct group in the Tukey test, demonstrating superior performance compared with the other treatments. In contrast, treatment P, which combined the highest digestate concentration with the highest water volume, resulted in the lowest flower production with only two flowers. This behavior is similar to that observed in experiment 1.

Regarding flower diameter, no significant differences were observed among treatments. According to Tukey’s test, all treatments were grouped together, indicating that neither the watering rate nor the digestate concentration significantly affected flower size in this experiment. Nevertheless, treatment J produced the largest flowers, with an average diameter of 53.41 mm.

Harvest time showed significant variation among treatments. Treatment P stood out, with the longest duration to harvest at 63 days, followed by treatment N, which took 62 days. These treatments formed a distinct group in Tukey’s test, suggesting that higher digestate concentrations may prolong the harvest period. However, besides reducing the final yield, a longer harvest period for only a few flowers hampers the efficient use of resources. In terms of flowering time, treatments K and D (232 mL of watering rate +0% digestate) achieved flowering later, at around 66 to 69 days after the experiment began, and formed a distinct group in Tukey’s test. In contrast, treatment N was the earliest to flower, at only 19 days, highlighting the potential accelerating effect of higher nutrient levels on reproductive development.^59,63^

The plant life cycle did not differ significantly across treatments, as all combinations of digestate and watering rate were grouped, indicating that these factors did not meaningfully affect the overall life cycle of pot marigold plants. The same behavior was observed in experiment 1.

Plant height exhibited significant differences among the treatments. Treatment F (118 mL of watering rate +10% digestate) produced the tallest plants at 17.93 cm, forming a distinct group in Tukey’s analysis. Other treatments with similar digestate concentrations, such as G and H, also performed better. In contrast, treatment P resulted in the shortest plants, at just 11.70 cm, suggesting that excessive input levels may hinder vertical growth.

The leaf area was positively influenced by certain treatments. Treatment G resulted in one of the largest leaf areas (420.4 cm^2^), forming a distinct group in Tukey analysis. Treatment P also performed well in this regard with 415.6 cm^2^ of leaf area. Meanwhile, treatments with the same water availability as that of treatment P, such as H, consistently produced smaller leaf areas, highlighting the benefit of a balanced watering rate and nutrient inputs for vegetative growth. This trend was also observed in experiment 1, where lower levels of digestate and water hindered leaf area development.

The fresh matter of the aerial part followed a similar pattern. Treatment L (232 mL of watering rate +20% digestate) yielded the highest fresh matter (21.93 g), indicating that these conditions promoted overall plant vigor. Treatment G also stood out with 21.19 g of fresh matter. In contrast, treatments with lower or excessive nutrient and water levels produced lower fresh matter, suggesting the importance of a balance between inputs.

The dry matter of the aerial part exhibited significant differences among treatments. Treatments G and O produced the highest dry matter, around 3.70 g, forming a distinct group in the Tukey test. These results demonstrate that moderate levels of water and digestate favor biomass accumulation.

Root dry matter was highest in treatment C (180 mL of watering rate +0% digestate) with 10.59 g, forming a distinct group in the Tukey analysis. This suggests that in this case, lower digestate concentration with adequate watering promotes optimal root development. Treatments with either excessive or insufficient nutrient levels tended to show a reduced root biomass.

In summary, treatments A and K were the most effective for increasing flower production. In contrast, treatment J produced the largest flowers, despite the lack of statistical differences in this parameter. Treatment M (57 mL of watering rate +30% digestate) stood out for extending the harvest time, while treatment F maximized the plant height. Treatment G consistently performed well, increasing the leaf area and fresh and dry matter of the aerial part. Finally, treatment C promoted the highest root dry weight.

In both experiment 1 (conducted during Brazil’s summer) and experiment 2 (conducted during Brazil’s winter), the influence of digestate concentration and watering rate on plant characteristics was apparent. However, significant differences between the two experiments emerged due to variations in environmental factors such as temperature, light, and humidity, which were influenced by the respective seasons.^64^ These environmental differences must be considered when comparing the results of the two experiments, as they likely played a role in shaping the observed plant responses.^65^

Both experiments demonstrated that moderate to high digestate concentrations (20–30%), combined with varying watering rates, increased flower production. In Experiment 1, Treatments H (16–18 flowers), I (16–18 flowers), and N (16–18 flowers) produced the most flowers, forming a distinct group in the Tukey test. In contrast, Experiment 2 had the most effective treatments in A and K (9 flowers), which, though effective, produced fewer flowers overall compared to the results of Experiment 1. The higher flower production in Experiment 1 can be attributed to the warmer temperatures, longer days, and higher humidity typical of Brazil’s summer months, which promoted accelerated plant growth and reproductive development. On the other hand, the cooler and shorter days of Experiment 2, which occurred during Brazil’s winter, likely contributed to slower growth, thereby reducing flower production despite the high nutrient levels.

In both experiments, higher digestate concentrations correlated with larger flowers. Experiment 2′s Treatment J (53.41 mm) produced the largest flowers, while in Experiment 1, Treatment P (56.67 mm) had the largest flowers, followed by Treatment K (55.53 mm). Although Experiment 1 produced slightly larger flowers overall, both experiments demonstrated that increased nutrient input resulted in larger flower sizes. The larger flowers in Experiment 1 may be attributed to the more favorable growth conditions (higher temperatures and longer daylight), which allowed plants to utilize the increased nutrient availability more effectively.

In experiment 1, there were no significant differences in harvest time across treatments, which might be due to the uniformity in environmental conditions that allowed plants to reach harvest maturity at similar rates. However, experiment 2 demonstrated a clear delay in harvest for treatments P (63 days) and N (62 days), indicating that higher nutrient concentrations, particularly in combination with larger watering volumes, delayed the harvest period. This delay was likely a result of the cooler temperatures and shorter days in experiment 2, which prolonged vegetative and reproductive stages. Similarly, flowering time in experiment 1 was achieved quickly in treatment P (60 days), while experiment 2 showed delayed flowering in treatments K and D (66–69 days). This suggests that the environmental conditions in experiment 1 accelerated flowering, while the cooler, less intense light conditions of experiment 2 delayed flower induction.

In Experiment 2, Treatment F produced the tallest plants (17.93 cm), suggesting that moderate water and digestate inputs promoted vertical growth under cooler conditions. In Experiment 1, there were no significant height differences, likely because the warmer temperatures and longer days allowed plants to grow more uniformly, regardless of the treatment. Both experiments demonstrated that leaf area and fresh matter of the aerial part were positively influenced by moderate to high concentrations of digestate. In Experiment 2, Treatment G resulted in the largest leaf area (420.4 cm^2^), while in Experiment 1, Treatment G also showed a large leaf area (400.5 cm^2^), but the fresh matter of the aerial part was higher (37.74 g). Treatment P in Experiment 2 showed a high leaf area (415.6 cm^2^) but had a significantly lower fresh matter of the aerial part (3.58 g), indicating that excessive nutrient and water levels may prioritize reproductive growth over vegetative mass accumulation. In contrast, Experiment 1’s more favorable conditions resulted in higher fresh matter across all treatments, suggesting that Experiment 1 provided a better environment for overall pot marigold plant vigor.

The dry matter of the aerial part did not show significant differences in either experiment, indicating that while the leaf area and fresh matter of the aerial part were affected by the treatments, the accumulation of dry matter remained relatively stable. However, the root dry matter showed distinct patterns. In experiment 2, treatment C produced the highest root dry matter (10.59 g), while in experiment 1, treatment J resulted in the highest root dry matter (16.99 g).

While both experiments demonstrated similar trends, such as higher digestate levels leading to improved flower production and larger flowers, experiment 1 generally produced more flowers and larger fresh matter, likely due to the warmer temperatures, longer daylight hours, and higher humidity during Brazil’s summer months. Treatment G stood out in both experiments for increasing leaf area and fresh matter of the aerial part, while treatment J was particularly effective for root development in experiment 1. However, experiment 2, conducted under cooler conditions, demonstrated that excessive nutrient levels (as observed in treatment P) resulted in delayed flowering and a prioritization of reproductive growth over vegetative growth. These differences underscore the importance of considering environmental factors when evaluating the impact of nutrient and water inputs on plant growth.

In conclusion, experiment 1 stands out as the more favorable environment for overall pot marigold growth and flower production, especially when combined with moderate nutrient levels and watering rates. Environmental conditions, such as temperature, light, and humidity, have a significant influence on plant growth and development. Therefore, future studies should consider these factors when designing experiments to optimize agricultural practices. The results underscore the need for a balanced approach in managing water and digestate levels to maximize plant performance under varying environmental conditions.

### 3.3 Pot Marigold Flower Characterization

Table 4 presents the physicochemical and bioactive composition of pot marigold flowers subjected to various water and digestate treatments in Experiment 1. Statistically significant differences (*p* ≤ 0.05) were observed among samples for all measured parameters, indicating that both inputs influenced flower composition.

**Table 4:** Physicochemical Determinations of Whole Flowers Experiment 1

	Physicochemical parameters^a^																
Sample	Dry matter (g 100 g^–1^)	Ash (g 100 g^–1^)	Nitrogen (g 100 g^–1^)	Protein (g 100 g^–1^)	Lipids (g 100 g^–1^)	Reducing sugars (g 100 g^–1^)	Nonreducing sugars (g 100 g^–1^)	Total sugars (g 100 g^–1^)	Chlorophyll a (mg 100 g^–1^)	Chlorophyll b (mg 100 g^–1^)	Total chlorophyll (mg 100 g^–1^)	TPC (mg GAE 100 g^–1^)	TFC (mg CE 100 g^–1^)	TCC (mg 100 g^–1^)	β-carotene (mg 100 g^–1^)	DPPH (μmol TEAC 100 g^–1^)	FRAP (μmol TEAC 100 g^–1^)
**A**	77.00 ± 0.79^ab^	7.03 ± 0.11^c^	1.09 ± 0.03^a^	6.80 ± 0.21^a^	5.59 ± 0.24^ab^	2.79 ± 0.19^a^	7.04 ± 0.21^d^	9.83 ± 0.49^d^	38.92 ± 0.88^cd^	23.43 ± 1.17^bc^	62.34 ± 0.51^c^	972.9 ± 40.68^abc^	147.9 ± 12.55^ab^	31.97 ± 0.21^a^	22.74 ± 0.24^a^	445.6 ± 5.44^abc^	650.9 ± 0.25^a^
**B**	75.32 ± 0.54^ab^	5.74 ± 0.22^b^	1.82 ± 0.11^abc^	11.40 ± 0.67^abc^	6.57 ± 0.55^abc^	2.60 ± 0.14^a^	7.62 ± 0.67^d^	10.22 ± 0.48^a^	28.22 ± 0.28^bc^	21.55 ± 0.29^bc^	49.75 ± 0.61^b^	1,069.5 ± 15.45^abcd^	173.7 ± 9.62^abcd^	61.59 ± 0.67^e^	40.60 ± 22.55^c^	552.8 ± 0.83^abcd^	704.2 ± 25.84^a^
**C**	74.01 ± 0.94^ab^	7.39 ± 0.12^c^	2.18 ± 0.14^bcd^	13.64 ± 0.90^bcd^	10.58 ± 0.43^c^	3.16 ± 0.24^a^	3.38 ± 0.90^ab^	6.54 ± 0.42^ab^	25.12 ± 0.25^b^	19.22 ± 0.08^b^	44.32 ± 0.69^b^	1,034.5 ± 75.00^abcd^	135.1 ± 0.77^ab^	47.24 ± 0.90^c^	31.59 ± 1.43^b^	345.0 ± 12.83^abc^	586.1 ± 6.34^a^
**D**	62.81 ± 0.19^a^	5.24 ± 0.21^b^	2.68 ± 0.03^abc^	16.74 ± 0.19^abc^	5.83 ± 0.18^ab^	2.65 ± 0.09^a^	5.04 ± 0.19^c^	7.69 ± 0.61^c^	22.08 ± 0.48^ab^	15.92 ± 0.89^ab^	37.99 ± 3.41^ab^	930.2 ± 63.41^abc^	163.7 ± 0.38^abc^	61.71 ± 0.19^e^	42.27 ± 0.18^c^	356.7 ± 18.33^abc^	791.4 ± 6.72^a^
**E**	73.79 ± 2.65^ab^	3.46 ± 0.09^a^	2.44 ± 0.20^bcd^	15.24 ± 0.25^bcd^	6.29 ± 0.35^abc^	3.45 ± 0.65^a^	2.98 ± 0.25^ab^	6.43 ± 0.11^ab^	28.87 ± 1.08^bc^	18.02 ± 1.03^b^	46.87 ± 3.03^b^	1,069.1 ± 53.64^abcd^	156.3 ± 2.55^ab^	66.90 ± 1.25^f^	46.34 ± 0.35^d^	233.9 ± 1.06^ab^	788.9 ± 1.66^a^
**F**	82.58 ± 0.17^b^	5.42 ± 0.10^b^	1.75 ± 0.06^abc^	10.91 ± 0.98^abc^	7.27 ± 0.67^bc^	2.16 ± 0.32^a^	4.48 ± 0.08^bc^	6.64 ± 0.30^ab^	17.33 ± 0.84^a^	12.30 ± 0.81^a^	29.62 ± 1.50^a^	1,136.8 ± 101.36^bcd^	215.5 ± 4.49^bcde^	62.59 ± 0.98^e^	42.35 ± 0.67^cd^	768.3 ± 7.06^cde^	849.4 ± 75.06^ab^
**G**	71.28 ± 0.44^ab^	3.69 ± 0.07^a^	1.36 ± 0.03^ab^	8.48 ± 0.18^ab^	3.46 ± 0.38^a^	2.22 ± 0.44^a^	4.22 ± 0.18^bc^	6.44 ± 0.34^ab^	14.44 ± 0.95^a^	8.20 ± 0.06^a^	22.63 ± 0.87^a^	919.3 ± 26.14^ab^	143.6 ± 1.54^ab^	42.25 ± 0.18^bc^	28.52 ± 0.38^b^	472.8 ± 6.28^abcd^	595.8 ± 17.72^a^
**H**	69.98 ± 0.76^ab^	4.15 ± 0.01^ab^	2.60 ± 0.15^cd^	16.22 ± 0.96^cd^	5.24 ± 0.23^ab^	1.90 ± 0.06^a^	4.95 ± 0.96^bc^	6.85 ± 0.01^ab^	46.85 ± 0.22^d^	29.83 ± 1.23^c^	76.66 ± 1.26^d^	803.9 ± 31.59^a^	88.46 ± 9.49^a^	46.25 ± 0.96^c^	30.20 ± 0.23^b^	320.6 ± 1.39^abc^	472.8 ± 8.00^a^
**I**	70.47 ± 3.69^ab^	4.37 ± 0.90^a^	2.84 ± 0.11^d^	17.77 ± 0.67^d^	4.73 ± 0.12^a^	2.56 ± 0.19^a^	4.96 ± 0.67^bc^	7.52 ± 0.90^bc^	40.15 ± 0.41^cd^	25.49 ± 0.46^bc^	65.63 ± 2.78^c^	1,012.5 ± 34.77^abcd^	265.8 ± 2.55^de^	55.86 ± 0.67^d^	39.55 ± 0.12^c^	607.8 ± 18.56^abcd^	836.9 ± 12.72^ab^
**J**	73.81 ± 4.44^ab^	6.48 ± 0.22^bc^	2.24 ± 0.24^bcd^	13.99 ± 1.53^bcd^	3.80 ± 0.16^a^	2.67 ± 0.44^a^	4.09 ± 0.53^ab^	6.76 ± 0.72^ab^	31.17 ± 0.78^c^	17.88 ± 0.87^b^	49.03 ± 0.23^b^	1,087.7 ± 31.36^abcd^	167.3 ± 8.08^abc^	41.32 ± 1.53^b^	29.39 ± 1.16^b^	147.8 ± 1.78^a^	862.3 ± 12.72^ab^
**K**	79.79 ± 0.82^ab^	2.84 ± 0.07^a^	2.69 ± 0.04^cd^	16.81 ± 0.22^cd^	2.23 ± 0.47^a^	1.77 ± 0.82^a^	4.55 ± 0.22^bc^	6.32 ± 0.69^ab^	30.52 ± 2.39^c^	21.44 ± 0.48^b^	51.94 ± 1.71^bc^	932.3 ± 37.73^abc^	265.1 ± 0.51^de^	63.75 ± 0.22^ef^	42.41 ± 0.47^cd^	987.2 ± 12.39^def^	1,031.6 ± 73.69^b^
**L**	68.33 ± 2.41^ab^	2.86 ± 0.06^b^	2.67 ± 0.29^cd^	16.67 ± 0.80^cd^	5.08 ± 0.03^ab^	3.04 ± 0.41^a^	4.44 ± 0.80^bc^	7.48 ± 0.25^bc^	42.89 ± 1.47^d^	27.15 ± 0.40^bc^	70.02 ± 1.08^cd^	943.6 ± 21.82^bcd^	220.8 ± 7.44^bcde^	124.8 ± 1.80^i^	80.48 ± 0.03^f^	732.8 ± 5.06^bcde^	825.6 ± 61.69^ab^
**M**	73.28 ± 3.32^ab^	6.24 ± 0.10^bc^	2.63 ± 0.01^cd^	16.47 ± 0.08^cd^	6.80 ± 0.07^abc^	4.43 ± 0.45^b^	2.76 ± 0.15^a^	7.19 ± 0.80^ab^	32.20 ± 0.25^c^	20.36 ± 0.33^b^	52.54 ± 3.27^bc^	1,285.4 ± 56.82^cd^	283.7 ± 8.33^abcde^	131.7 ± 0.08^h^	80.97 ± 1.07^f^	1,334.4 ± 13.22^abcd^	1,255.8 ± 61.28^b^
**N**	72.95 ± 1.23^ab^	2.96 ± 0.13^a^	2.31 ± 0.46^bcd^	14.43 ± 2.88^bcd^	6.77 ± 0.02^abc^	3.70 ± 0.23^b^	3.93 ± 0.88^ab^	7.63 ± 0.13^bc^	25.25 ± 0.47^b^	22.44 ± 0.71^bc^	47.68 ± 3.71^b^	1,222.7 ± 13.18^d^	255.00 ± 11.41^e^	125.4 ± 2.88^hi^	82.18 ± 0.02^f^	1,225.5 ± 4.56^f^	1,154.5 ± 38.66^b^
**O**	76.22 ± 1.49^ab^	5.43 ± 0.17^b^	2.37 ± 0.10^bcd^	14.83 ± 2.51^bcd^	6.39 ± 0.55^abc^	2.49 ± 0.49^a^	4.07 ± 0.51^ab^	6.56 ± 1.17^ab^	24.06 ± 0.44^b^	17.28 ± 0.65^b^	41.32 ± 7.53^b^	1,192.9 ± 13.41^bcd^	161.8 ± 2.31^cde^	99.84 ± 2.51^g^	68.90 ± 0.55^e^	612.8 ± 2.61^abcd^	974.8 ± 42.16^b^
**P**	77.14 ± 3.37^ab^	6.13 ± 0.20^bc^	2.11 ± 0.03^bcd^	13.21 ± 0.20^bcd^	5.60 ± 0.26^ab^	2.92 ± 0.37^a^	4.20 ± 0.20^bc^	7.12 ± 0.18^ab^	39.91 ± 1.42^cd^	25.12 ± 0.25^bc^	65.01 ± 1.39^c^	1,143.6 ± 84.09^bcd^	204.4 ± 3.85^bcde^	101.2 ± 0.20^g^	67.07 ± 0.26^e^	785.6 ± 2.44^cde^	961.2 ± 13.13^b^

a Results expressed as the mean ± standard deviation of triplicates. Different lowercase letters indicate significant differences among treatments based on Tukey’s test at *p* ≤ 0.05. Sample codes: A: 57 mL water and 0% digestate; B: 118 mL water and 0% digestate; C: 180 mL water and 0% digestate; D: 232 mL water and 0% digestate; E: 57 mL water and 10% digestate; F: 118 mL water and 10% digestate; G: 180 mL water and 10% digestate; H: 232 mL water and 10% digestate; I: 57 mL water and 20% digestate; J: 118 mL water and 20% digestate; K: 180 mL water and 20% digestate; L: 232 mL water and 20% digestate; M: 57 mL water and 30% digestate; N: 118 mL water and 30% digestate; O: 180 mL water and 30% digestate; P: 232 mL water and 30% digestate.

Dry matter content exhibited apparent differences, with sample D (232 mL watering rate +0% digestate) having the lowest value (62.81 g 100 g^–1^), reflecting the dilution effect of a high water input in the absence of digestate. In contrast, sample F (118 mL of watering rate +10% digestate) showed the highest dry matter (82.58 g 100 g^–1^), suggesting that moderate digestate levels can enhance the biomass concentration. Higher dry matter is desirable in food and nutraceutical processing as it facilitates storage, reduces spoilage risk, and concentrates valuable compounds.^20^

Ash content, a proxy for total mineral content, peaked in sample C (180 mL watering rate +0% digestate) at 7.39 g 100 g^–1^. However, the lowest ash value was observed in sample K (180 mL of watering rate +20% digestate), suggesting that higher digestate levels may not necessarily enhance mineral accumulation.

Nitrogen and protein contents were significantly influenced by digestate input. Sample I (57 mL of watering rate +20% digestate) showed the highest nitrogen (2.84 g 100 g^–1^) and protein (17.77 g 100 g^–1^). These values suggest strong potential for protein-enriched applications of pot marigold flowers grown under these conditions. High-protein floral biomass could serve as an ingredient in functional flours, feed additives, or plant-based protein supplements, especially considering the increasing demand for sustainable, botanical protein sources.^66,67^

Lipids were highest in sample C (10.58 g 100 g^–1^), highlighting the value of a moderate watering rate in enhancing lipid accumulation. Lipids in pot marigold flowers often include essential fatty acids and bioactive lipid-soluble compounds, supporting their application in cosmetics, skin-nourishing formulations, and nutraceutical oil extracts.^68^

In terms of carbohydrates, sample M (57 mL of watering rate +30% digestate) exhibited the highest reducing (4.43 g 100 g^–1^) and nonreducing sugars (2.76 g 100 g^–1^), resulting in the highest total sugars. Carbohydrate-rich pot marigold extracts could be relevant for developing energy-boosting infusions, syrups, or functional food blends.^69^ The sugar profile also contributes to taste and potential prebiotic properties.^70^

The chlorophyll content revealed differentiated effects. Samples with no digestate (e.g., A) had the highest chlorophyll a (38.92 mg 100 g^–1^) and b (23.43 mg 100 g^–1^), while those with excess water and low digestate (e.g., G: 180 mL of watering rate +10% digestate) had the lowest. This indicates that while digestate supports other quality attributes, chlorophyll synthesis may be hampered at higher nutrient loads, possibly due to nitrogen-induced chlorophyll degradation or changes in plant stress signaling.^71^

Carotenoids, including β-carotene, showed a positive response to digestate, with sample M reaching 131.7 mg 100 g^–1^ TCC and 80.97 mg 100 g^–1^ of β-carotene. These are particularly important for human health, as β-carotene is a precursor to vitamin A and a potent antioxidant. Pot marigold flowers with high carotenoid content are thus ideal for use in dietary supplements, skin-repair ointments, and natural food colorants.^72,73^

Phenolic compounds and flavonoids increased with digestate application, with sample M showing peak total phenolics (1285.4 mg of GAE 100 g^–1^) and sample K showing the highest flavonoids (265.8 mg of CE 100 g^–1^). These compounds are key contributors to antioxidant, anti-inflammatory, and antimicrobial activities.^22^ Pot marigold flowers cultivated under these conditions can be ideal for use in herbal teas, topical salves, and as active agents in antiaging and therapeutic cosmetic formulations.^69^

Lastly, antioxidant activity, assessed through DPPH and FRAP assays, correlated with the levels of phenolics and flavonoids. Sample M had the highest values in both assays (DPPH: 1334.4 μmol TEAC 100 g^–1^; FRAP: 1255.8 μmol TEAC 100 g^–1^). This high antioxidant potential supports the functional use of these flowers in managing oxidative stress, dietary antioxidant supplementation, and natural preservative systems.^74^

Table 5 details the physicochemical and biochemical properties of pot marigold flowers for experiment 2.

**Table 5:** Physicochemical Determinations of Whole Flowers Experiment 2

	Physicochemical parameters^a^																
Sample	Dry matter (g 100 g^–1^)	Ash (g 100 g^–1^)	Nitrogen (g 100 g^–1^)	Protein (g 100 g^–1^)	Lipids (g 100 g^–1^)	Reducing sugars (g 100 g^–1^)	Nonreducing sugars (g 100 g^–1^)	Total sugars (g 100 g^–1^)	Chlorophyll a (mg 100 g^–1^)	Chlorophyll b (mg 100 g^–1^)	Total chlorophyll (mg 100 g^–1^)	TPC (mg GAE 100 g^–1^)	TFC (mg CE 100 g^–1^)	TCC (mg 100 g^–1^)	β-carotene (mg 100 g^–1^)	DPPH (μmol TEAC 100 g^–1^)	FRAP (μmol TEAC 100 g^–1^)
**A**	81.08 ± 0.51^b^	5.98 ± 0.11^b^	1.04 ± 0.14^a^	6.51 ± 0.88^a^	6.68 ± 0.17^ab^	2.63 ± 0.11^ab^	3.46 ± 0.28^b^	6.31 ± 0.41^a^	40.00 ± 0.21^bc^	23.13 ± 0.24^b^	63.12 ± 0.79^c^	935.9 ± 25.91^abc^	56.03 ± 1.64^a^	72.04 ± 0.51^de^	51.65 ± 0.41^def^	214.4 ± 3.44^ab^	604.8 ± 8.66^a^
**B**	75.99 ± 0.61^ab^	7.40 ± 0.17^bc^	2.16 ± 0.04^b^	13.52 ± 0.28^b^	8.09 ± 0.29^bc^	3.30 ± 0.61^b^	3.29 ± 0.28^b^	6.80 ± 0.37^ab^	28.88 ± 0.67^b^	22.40 ± 2.55^b^	51.27 ± 0.54^bc^	1,022.5 ± 95.68^bc^	142.3 ± 8.97^abcde^	62.93 ± 0.61^b^	42.04 ± 0.37^b^	184.4 ± 2.22^ab^	735.2 ± 31.66^a^
**C**	81.59 ± 0.69^b^	6.91 ± 0.28^bc^	1.99 ± 0.13^ab^	12.43 ± 0.84^ab^	7.32 ± 0.08^b^	3.36 ± 0.69^b^	3.33 ± 0.25^b^	6.90 ± 0.38^ab^	26.09 ± 0.90^b^	20.06 ± 1.43^b^	46.14 ± 0.94^b^	1,124.5 ± 46.84^bc^	185.9 ± 17.44^bcde^	35.31 ± 0.69^a^	23.77 ± 0.38^a^	615.6 ± 2.67^cd^	688.7 ± 14.25^a^
**D**	79.96 ± 3.41^ab^	8.39 ± 0.31^c^	2.19 ± 0.15^b^	13.70 ± 0.95^b^	8.13 ± 0.89^bc^	4.06 ± 0.41^c^	2.35 ± 0.48^a^	6.56 ± 0.11^a^	22.96 ± 0.19^ab^	16.55 ± 0.18^ab^	39.50 ± 0.19^b^	1,116.6 ± 13.41^bc^	167.9 ± 7.18^bcde^	78.62 ± 3.41^f^	53.27 ± 0.11^f^	113.9 ± 5.39^a^	781.7 ± 12.28^a^
**E**	66.08 ± 3.03^ab^	5.29 ± 0.07^b^	2.78 ± 0.17^d^	17.37 ± 0.08^d^	8.35 ± 0.03^c^	4.09 ± 0.03^c^	3.80 ± 0.38^b^	8.13 ± 0.03^c^	29.66 ± 1.25^b^	18.98 ± 0.35^b^	48.62 ± 2.65^b^	933.6 ± 61.36^abc^	126.3 ± 3.46^abc^	75.59 ± 3.03^ef^	52.33 ± 0.03^ef^	535.6 ± 2.33^cd^	634.5 ± 35.91^a^
**F**	68.55 ± 1.50^ab^	6.94 ± 0.08^bc^	2.25 ± 0.04^b^	14.05 ± 0.25^b^	12.30 ± 0.81^d^	3.00 ± 0.50^b^	4.29 ± 0.24^ab^	7.56 ± 0.48^b^	24.77 ± 0.98^b^	17.51 ± 0.67^ab^	42.27 ± 0.17^b^	1,006.8 ± 67.73^abc^	122.6 ± 2.31^abc^	36.49 ± 1.50^a^	23.82 ± 0.48^a^	382.2 ± 9.33^bc^	901.7 ± 83.66^a^
**G**	67.35 ± 0.87^ab^	2.48 ± 0.10^a^	2.12 ± 0.08^b^	13.28 ± 0.48^b^	5.62 ± 0.06^ab^	4.02 ± 0.87^c^	2.96 ± 0.25^a^	7.17 ± 0.19^b^	18.43 ± 0.18^a^	14.57 ± 0.38^ab^	32.99 ± 0.44^ab^	941.8 ± 38.64^abc^	133.2 ± 5.77^abcd^	68.85 ± 0.87^cde^	46.19 ± 0.19^cd^	395.6 ± 0.56^bc^	606.8 ± 9.06^a^
**H**	60.53 ± 1.26^a^	4.22 ± 0.19^ab^	2.20 ± 0.04^b^	13.73 ± 0.22^b^	7.43 ± 0.23^ab^	3.04 ± 0.26^b^	6.13 ± 0.22^c^	9.57 ± 0.29^d^	14.96 ± 0.96^a^	10.72 ± 0.23^a^	25.67 ± 0.76^a^	680.9 ± 15.91^a^	108.1 ± 0.38^ab^	72.21 ± 1.26^ef^	48.85 ± 0.29^de^	162.8 ± 6.17^ab^	425.2 ± 5.41^a^
**I**	76.71 ± 2.78^ab^	4.57 ± 0.11^ab^	2.71 ± 0.07^c^	16.91 ± 0.41^c^	7.74 ± 0.46^ab^	3.06 ± 0.78^b^	4.00 ± 0.41^ab^	7.31 ± 0.08^b^	48.97 ± 0.67^c^	30.43 ± 0.12^c^	79.37 ± 3.69^d^	925.4 ± 68.64^abc^	181.8 ± 5.90^bcde^	72.31 ± 2.78^ef^	48.94 ± 0.08^de^	546.1 ± 7.83^cd^	1,105.5 ± 45.59^a^
**J**	75.46 ± 0.23^ab^	4.01 ± 0.13^ab^	1.86 ± 0.13^ab^	10.59 ± 0.78^ab^	4.95 ± 0.87^a^	2.85 ± 0.23^ab^	3.70 ± 0.78^ab^	6.79 ± 0.23^ab^	41.45 ± 1.53^c^	26.35 ± 1.16^bc^	67.78 ± 4.44^c^	1,025.4 ± 40.91^bc^	122.0 ± 2.55^abc^	59.00 ± 0.23^b^	40.86 ± 0.23^b^	889.4 ± 2.72^ef^	751.1 ± 18.47^a^
**K**	75.16 ± 1.71^ab^	6.61 ± 0.30^ab^	2.11 ± 0.38^b^	13.20 ± 1.39^b^	4.65 ± 0.48^a^	1.91 ± 0.11^a^	4.07 ± 0.39^ab^	6.24 ± 0.40^a^	31.40 ± 0.22^b^	18.02 ± 0.47^ab^	49.41 ± 0.82^b^	806.8 ± 66.82^ab^	116.1 ± 2.56^abc^	67.93 ± 1.71^cd^	44.28 ± 1.40^bc^	165.0 ± 2.61^ab^	962.8 ± 24.19^a^
**L**	67.72 ± 1.08^ab^	6.34 ± 0.22^bc^	2.42 ± 0.23^bc^	15.15 ± 1.47^bc^	4.80 ± 0.40^a^	4.03 ± 0.08^c^	3.05 ± 0.47^b^	7.28 ± 0.30^b^	31.71 ± 1.80^b^	21.34 ± 0.03^b^	53.04 ± 2.41^bc^	1,172.5 ± 15.68^c^	215.9 ± 15.69^de^	66.19 ± 1.08^c^	44.48 ± 1.30^bc^	702.8 ± 5.50^de^	1,065.3 ± 28.63^a^
**M**	76.27 ± 3.27^ab^	3.07 ± 0.04^ab^	2.68 ± 0.03^c^	16.75 ± 0.25^c^	7.81 ± 0.33^ab^	2.68 ± 0.27^ab^	4.50 ± 0.25^ab^	7.48 ± 0.74^b^	44.26 ± 0.08^c^	29.14 ± 1.07^c^	73.38 ± 3.32^d^	944.1 ± 28.64^abc^	202.8 ± 5.13^cde^	118.0 ± 3.27^j^	78.40 ± 0.74^i^	596.1 ± 1.72^cd^	1,277.5 ± 80.53^a^
**N**	69.99 ± 3.71^ab^	6.91 ± 0.19^bc^	2.22 ± 0.08^b^	13.87 ± 0.47^b^	8.32 ± 0.71^bc^	3.39 ± 0.71^b^	3.97 ± 0.47^ab^	7.62 ± 0.39^b^	32.72 ± 2.88^b^	21.35 ± 0.02^b^	54.05 ± 1.23^bc^	1,217.9 ± 92.05^c^	227.0 ± 13.21^e^	128.4 ± 2.88^k^	88.33 ± 0.02^j^	1,002.2 ± 4.56^f^	1,314.8 ± 29.00^a^
**O**	79.20 ± 7.53^ab^	2.33 ± 0.03^a^	2.13 ± 0.07^b^	13.32 ± 0.44^b^	7.65 ± 0.65^ab^	2.82 ± 0.13^ab^	3.77 ± 0.44^ab^	6.84 ± 0.04^ab^	25.71 ± 2.51^b^	25.07 ± 0.55^bc^	50.76 ± 1.49^bc^	912.9 ± 13.41^abc^	121.5 ± 10.00^abc^	86.36 ± 7.53^h^	59.37 ± 0.04^g^	627.5 ± 5.28^cde^	654.5 ± 17.91^a^
**P**	80.97 ± 1.39^b^	5.93 ± 0.22^ab^	2.21 ± 0.32^b^	13.83 ± 1.42^b^	7.38 ± 0.25^ab^	2.65 ± 0.39^ab^	3.20 ± 0.42^ab^	6.06 ± 0.30^a^	38.68 ± 0.20^bc^	24.48 ± 0.26^bc^	63.14 ± 3.37^c^	1,046.4 ± 34.09^bc^	168.7 ± 18.46^bcde^	100.2 ± 0.20^i^	65.33 ± 0.26^h^	111.7 ± 1.72^a^	1,091.9 ± 43.13^a^

a Results expressed as the mean ± standard deviation of triplicates. Different lowercase letters indicate significant differences among treatments based on Tukey’s test at *p* ≤ 0.05. Sample codes: A: 57 mL water and 0% digestate; B: 118 mL water and 0% digestate; C: 180 mL water and 0% digestate; D: 232 mL water and 0% digestate; E: 57 mL water and 10% digestate; F: 118 mL water and 10% digestate; G: 180 mL water and 10% digestate; H: 232 mL water and 10% digestate; I: 57 mL water and 20% digestate; J: 118 mL water and 20% digestate; K: 180 mL water and 20% digestate; L: 232 mL water and 20% digestate; M: 57 mL water and 30% digestate; N: 118 mL water and 30% digestate; O: 180 mL water and 30% digestate; P: 232 mL water and 30% digestate.

The dry matter content varied considerably among treatments. Sample C exhibited the highest dry matter concentration (81.59 g 100 g^–1^), significantly surpassing samples such as H (232 mL of watering rate +10% digestate) (60.53 g 100 g^–1^), suggesting more concentrated biomass under moderate water regimes. Higher dry matter is favorable for storage stability, extractability, and downstream processing. Sample G, despite its intermediate digestate concentration, had one of the lowest dry matter values (63.87 g 100 g^–1^), indicating that excessive water inputs in combination with moderate digestate may suppress solid accumulation in floral tissues.

Ash content, indicative of total mineral load, ranged from 2.33 g 100 g^–1^ (O: 180 mL of watering rate +30% digestate) to 8.39 g 100 g^–1^ (D), a 4-fold variation across treatments. The high ash content in sample D suggests enhanced mineral uptake, possibly due to efficient nutrient absorption under high watering level conditions and a low digestate proportion. The clear distinction with sample O again highlights the importance of a balanced digestate in maximizing nutrient assimilation into floral tissues.

Nitrogen and protein contents, which are central to nutritional and functional evaluations, showed consistent trends. Sample E (57 mL of watering rate +10% digestate) registered the highest nitrogen (2.78 g 100 g^–1^) and protein (17.37 g 100 g^–1^) levels, statistically distinct from samples like A (57 mL of watering rate +0% digestate) (1.04 and 6.51 g 100 g^–1^, respectively). These results highlight the crucial role of a high digestate concentration in promoting amino acid synthesis and protein accumulation. Such profiles may enhance the value of flower biomass for nutraceutical and feed applications. Conversely, sample A, with low digestate and low water input, showed the poorest nitrogenous profile, highlighting the need for nutrient-rich conditions in protein-focused production strategies.

The lipid content was similarly influenced by treatment combinations. Sample F exhibited the highest value (12.30 g 100 g^–1^), significantly greater than that of K (4.65 g 100 g^–1^). Interestingly, the lipid content did not always correlate with protein levels, suggesting distinct metabolic pathways responsive to nutrient stress or sufficiency. Lipid-rich samples such as E, F, and N (118 mL of watering rate +30% digestate) may hold promise for the extraction of essential oils or formulation into cosmetic products where lipid-soluble compounds are desired.

Carbohydrate profiles, measured via reducing and total sugars, presented moderate variation. Reducing sugars peaked in sample E (4.09 g 100 g^–1^), followed closely by samples D and L (232 mL of watering rate +20% digestate). These sugars contribute to energy content, taste, and potential prebiotic activity. The high sugar content in D complements its high lipid and mineral profile, further affirming its suitability for multifunctional applications.

Pigment concentrations, especially chlorophylls and carotenoids, were markedly treatment-dependent. Sample I led in total chlorophylls (79.37 mg 100 g^–1^), chlorophyll a (63.60 mg 100 g^–1^), and chlorophyll b (15.77 mg 100 g^–1^), indicating high photosynthetic and metabolic activity within the floral tissues, likely due to a favorable balance of digestate and water. These pigments are valued not only for coloration but also for their antioxidant and anti-inflammatory activities.^73^ High pigment content enhances the flower’s appeal for natural dye production and therapeutic formulations.

The total phenolic content reached its highest in sample L (1172.5 mg of GAE 100 g^–1^), with several other samples (e.g., M, N, and I) also showing elevated phenolic levels above 1000 mg of GAE 100 g^–1^. These compounds are primary antioxidants and play a protective role in oxidative stress. High-phenolic flowers are particularly valuable for incorporation into health-promoting food or cosmetic products. Total flavonoids showed a similar trend, peaking in sample N (227.0 mg of CE 100 g^–1^), which also had high FRAP and DPPH values, confirming a strong antioxidant profile.

Carotenoids, including total carotenoids and β-carotene, were significantly elevated in sample M (118.0 mg 100 g^–1^), indicating exceptional provitamin A potential. These lipophilic pigments are essential for eye health and skin protection and are widely used in nutraceuticals and supplements. Samples M and N thus emerge as top candidates for functional applications centered on antioxidant and anti-inflammatory effects.

Finally, antioxidant capacity, as assessed by DPPH and FRAP assays, confirmed the biochemical richness of these floral tissues. Sample N displayed the highest DPPH scavenging activity (1002.2 μmol TEAC 100 g^–1^), while FRAP values peaked in N (1314.8 μmol TEAC 100 g^–1^) and M (1277.5 μmol TEAC 100 g^–1^), corroborating the phenolic and flavonoid data. These findings emphasize the suitability of these treatments for producing high-antioxidant floral biomass with potential therapeutic value.

In summary, *C. officinalis* L. “Pôr do Sol” flowers responded strongly to fertilization with digestate, particularly at 20–30% levels. Their nutritional (protein, lipid, and sugar) and bioactive (phenolic, carotenoid, and antioxidant) profiles suggest diverse application potential across the food, feed, cosmetic, and pharmaceutical sectors. Tailoring water and digestate inputs enables the optimization of desired compound classes according to end-use priorities.

Experiments 1 and 2 evaluated the effects of irrigation and digestate concentrations on the chemical composition of pot marigold flowers during Brazil’s summer and winter, respectively. Both showed statistically significant treatment effects (*p* ≤ 0.05), with seasonal contrasts driven by temperature, solar radiation, and evapotranspiration differences that affect plant metabolism.

Dry matter accumulation was seasonally distinct. In summer (experiment 1), sample F had the highest dry matter (82.58 g 100 g^–1^), whereas excess water without digestate (sample D) caused dilution. In winter (experiment 2), dry matter also peaked under moderate irrigation (sample C), but values were slightly lower, likely due to reduced evapotranspiration. Despite lower winter mineral uptake efficiency, ash content was higher in winter samples (e.g., D: 33.45 g 100 g^–1^) than in summer (e.g., F: 25.70 g 100 g^–1^), reflecting slower growth and mineral retention.

Digestate enhanced nitrogen and protein levels across seasons. The highest protein in summer (17.77 g 100 g^–1^, sample I) exceeded winter’s peak (17.37 g 100 g^–1^, sample E), likely due to higher summer metabolic activity promoting nitrogen assimilation.

The lipid content was high in both seasons. Summer sample C (10.58 g 100 g^–1^) showed strong lipid synthesis, possibly due to heat-induced production of protective lipids. Winter sample F also had high lipids (12.30 g 100 g^–1^), suggesting nutrient-driven lipid metabolism across seasons.

Sugar content was higher in summer, especially in sample M (4.43 g of 100 g^–1^ reducing sugars), reflecting enhanced photosynthesis. Winter values, while still notable (e.g., sample E: 4.09 g 100 g^–1^), were lower, likely due to cooler temperatures and reduced carbon assimilation.

Pigment content varied strongly with season. Summer chlorophyll levels peaked under low digestate (sample A), potentially due to stress-related retention, while winter saw overall higher pigment concentrations, with sample I reaching 79.37 mg 100 g^–1^ total chlorophylls. Carotenoids, especially β-carotene, also responded well to digestate, with summer sample M showing the highest levels (131.7 mg 100 g^–1^ TCC; 80.97 mg 100 g^–1^ β-carotene), although winter values were slightly lower.

Phenolics and flavonoids increased with the addition of the digestate in both seasons. Sample M (summer) had the highest TPC (1285.4 mg of GAE 100 g^–1^), followed by winter sample L (1172.5 mg of GAE 100 g^–1^). Antioxidant activity (DPPH and FRAP) was also high in both periods, with the summer sample M and winter sample N achieving the strongest values, indicating that digestate promotes the accumulation of bioactive compounds regardless of the season, with cooler temperatures possibly aiding in the preservation of these compounds.

In conclusion, tailoring water and digestate inputs to seasonal conditions can optimize the quality of pot marigold flowers. Summer favored higher synthesis of sugar, protein, and carotenoids, while winter enhanced pigment retention and mineral accumulation. Digestate at 20–30% consistently boosted key compounds, and irrigation modulated biomass and mineral dilution. These findings support the use of digestate for producing high-quality flowers for functional foods, cosmetics, and nutraceuticals within sustainable, circular bioeconomy frameworks.

Tables S3 and S4 show the results for the color parameters of the pot marigold flower extracts. The CIELab color parameters revealed differences among samples (A–P), despite the overall similarity in hue angle (H°), which remained consistently close to 98.6° across all treatments. This indicates that the base hue, a yellowish-green tone, was largely preserved, and that perceived color differences.

In terms of lightness (L*), values ranged from 92.19 to 96.57. Most samples exhibited high brightness, yet sample K was slightly brighter than the others, while sample O appeared slightly darker, suggesting a modest reduction in reflectance that may point to increased pigment accumulation or changes in the surface structure. Regarding the red-green axis (a*), all samples fell in the green spectrum (negative values) but with varying intensity. Samples G, H, and I showed noticeably higher green tones, with sample G exhibiting the most pronounced greens. In contrast, samples E, K, and M were slightly light green, leaning closer to neutral, which may indicate an enhanced presence of competing hues.

On the yellow-blue axis (b*), values spanned from 22.49 to 38.99. Samples I and O were distinctly more yellow than the others, giving them a warmer and richer appearance. Conversely, sample K presented a moderately reduced yellow component, resulting in a paler tone. These differences were further emphasized by chroma (C*), where samples I and O again stood out due to their moderately higher saturation levels, suggesting a more vivid and vibrant color expression. Sample K, on the other hand, exhibited noticeably lower chroma, corresponding to a muted and less intense visual impression.

Overall, sample G stood out for its vibrant green-yellow coloration, while sample I was among the most saturated and vividly colored with a yellow hue. Sample K consistently showed reduced chroma, lightness, and yellow intensity, resulting in a less vivid and paler appearance. Meanwhile, sample O combined slightly lower lightness with higher yellowness and chroma, producing a deeper, warmer hue. Despite these variations, the stable hue angle across all samples confirms that the core pigment profile remained within a narrow chromatic range with subtle shifts primarily driven by changes in saturation and brightness.

The fatty acid profiles of the pot marigold flower samples are presented in Table 6. Saturated fatty acids (SFAs) varied widely, from 98.26% in sample I (57 mL of watering +20% digestate) to just 7.51% in sample A. Samples C, I, and J (118 mL of watering +20% digestate), which exhibited the highest SFA levels, differed significantly from samples A and B (118 mL of watering +0% digestate), which showed the lowest SFA content. Elevated consumption of saturated fats has been associated with higher LDL cholesterol and increased cardiovascular risk.^75^ Consequently, the lower SFA levels observed in some samples indicate more favorable nutritional quality and potential health benefits.^76^

**Table 6:** Fatty Acids Composition of Pot Marigold Flowers^a^

	Pot marigold flower samples															
Fatty acids (%)	A	B	C	D	E	F	G	H	I	J	K	L	M	N	O	P
C4:0	nd	nd	10.89 ± 0.921	17.49 ± 1.003	6.882 ± 0.750	17.63 ± 1.030	49.14 ± 3.443	35.98 ± 2.110	81.49 ± 3.578	68.60 ± 2.550	56.29 ± 3.101	45.22 ± 1.111	42.78 ± 3.335	nd	23.65 ± 0.957	48.10 ± 1.550
C6:0	0.545 ± 0.030	0.696 ± 0.052	1.368 ± 0.330	1.100 ± 0.090	0.875 ± 0.022	nd	0.886 ± 0.051	0.534 ± 0.031	0.733 ± 0.066	1.036 ± 0.133	0.985 ± 0.077	0.846 ± 0.031	0.722 ± 0.022	1.143 ± 0.131	nd	0.851 ± 0.044
C8:0	nd	6.094 ± 0.230	57.76 ± 4.271	28.79 ± 1.113	49.36 ± 5.270	49.17 ± 3.333	8.152 ± 0.915	1.754 ± 0.055	3.904 ± 0.431	nd	1.937 ± 0.033	4.794 ± 0.133	3.467 ± 0.230	59.81 ± 1.330	16.81 ± 0.530	3.399 ± 0.067
C10:0	0.319 ± 0.020	0.549 ± 0.030	1.637 ± 0.210	1.026 ± 0.066	1.307 ± 0.051	2.670 ± 0.123	0.376 ± 0.010	0.309 ± 0.012	0.848 ± 0.044	0.419 ± 0.011	0.404 ± 0.022	0.359 ± 0.009	0.358 ± 0.014	4.148 ± 0.178	0.272 ± 0.011	0.374 ± 0.017
C11:0	nd	0.237 ± 0.013	0.453 ± 0.021	0.526 ± 0.045	0.382 ± 0.017	0.450 ± 0.077	0.255 ± 0.015	0.239 ± 0.022	0.252 ± 0.020	0.272 ± 0.034	0.241 ± 0.024	0.238 ± 0.061	0.244 ± 0.010	0.414 ± 0.051	0.229 ± 0.011	0.241 ± 0.010
C12:0	nd	0.326 ± 0.047	0.353 ± 0.015	0.317 ± 0.021	0.270 ± 0.011	0.293 ± 0.020	0.459 ± 0.037	0.285 ± 0.063	nd	nd	0.370 ± 0.051	0.297 ± 0.018	0.494 ± 0.029	0.798 ± 0.080	0.312 ± 0.021	0.387 ± 0.033
C13:0	0.262 ± 0.022	0.284 ± 0.020	1.167 ± 0.097	0.732 ± 0.055	0.666 ± 0.021	0.868 ± 0.033	1.067 ± 0.105	0.911 ± 0.087	1.222 ± 0.212	1.497 ± 0.133	1.250 ± 0.105	1.113 ± 0.074	0.846 ± 0.055	0.984 ± 0.066	0.572 ± 0.021	1.070 ± 0.078
C14:0	nd	nd	1.438 ± 0.130	0.781 ± 0.059	0.679 ± 0.024	0.976 ± 0.033	2.314 ± 0.230	1.189 ± 0.160	2.511 ± 0.188	1.806 ± 0.121	2.043 ± 0.330	1.548 ± 0.055	1.405 ± 0.014	0.213 ± 0.022	0.835 ± 0.031	1.665 ± 0.111
C14:1	0.192 ± 0.018	0.197 ± 0.009	4.109 ± 0.583	2.624 ± 0.111	1.959 ± 0.083	2.775 ± 0.178	3.675 ± 0.236	4.264 ± 0.455	nd	7.714 ± 0.743	5.369 ± 0.515	5.370 ± 0.445	3.456 ± 0.136	0.558 ± 0.051	nd	nd
C15:0	nd	nd	0.190 ± 0.008	nd	nd	nd	nd	nd	0.225 ± 0.012	nd	0.186 ± 0.022	nd	0.240 ± 0.031	2.078 ± 0.121	2.486 ± 0.222	1.585 ± 0.080
C15:1	0.630 ± 0.215	0.605 ± 0.184	0.644 ± 0.155	0.498 ± 0.089	0.969 ± 0.112	nd	nd	nd	nd	nd	nd	nd	nd	1.227 ± 0.333	nd	nd
C16:0	nd	nd	1.690 ± 0.120	0.819 ± 0.055	0.926 ± 0.082	nd	1.586 ± 0.101	0.949 ± 0.066	2.042 ± 0.221	nd	1.892 ± 0.077	1.599 ± 0.066	1.875 ± 0.051	nd	4.158 ± 0.222	2.656 ± 0.101
C16:1	nd	0.453 ± 0.023	nd	nd	nd	nd	nd	nd	0.463 ± 0.054	0.454 ± 0.041	0.458 ± 0.031	nd	nd	nd	nd	nd
C17:0	nd	0.559 ± 0.015	0.509 ± 0.030	0.474 ± 0.047	nd	nd	0.961 ± 0.014	nd	nd	0.675 ± 0.022	nd	0.605 ± 0.044	0.595 ± 0.051	nd	nd	0.854 ± 0.040
C17:1	0.192 ± 0.00	nd	nd	nd	nd	nd	nd	nd	nd	nd	nd	nd	nd	0.355 ± 0.021	2.704 ± 0.455	1.323 ± 0.054
C18:0	0.570 ± 0.011	nd	2.322 ± 0.214	nd	nd	1.494 ± 0.070	0.487 ± 0.022	2.314 ± 0.077	nd	nd	2.739 ± 0.133	nd	1.363 ± 0.044	nd	nd	2.003 ± 0.137
C18:1(*trans*)	nd	nd	nd	nd	nd	nd	nd	nd	nd	0.723 ± 0.055	nd	nd	nd	nd	nd	nd
C18:1(*cis*)	2.997 ± 0.222	3.793 ± 0.410	nd	nd	nd	nd	nd	0.578 ± 0.055	nd	nd	nd	nd	nd	nd	nd	nd
C18:2(*trans*)	26.18 ± 5.150	nd	nd	18.09 ± 3.212	nd	nd	nd	nd	nd	nd	nd	nd	nd	nd	nd	nd
C18:2(*cis*)	nd	36.94 ± 4.111	nd	nd	20.01 ± 2.008	nd	nd	nd	nd	nd	nd	nd	nd	17.70 ± 3.480	12.40 ± 2.690	nd
C18:3(γ)	27.41 ± 3.188	nd	nd	nd	nd	nd	0.825 ± 0.041	nd	nd	nd	nd	nd	nd	nd	nd	nd
C18:3(α)	nd	nd	nd	1.262 ± 0.022	1.321 ± 0.047	nd	1.475 ± 0.058	1.893 ± 0.012	2.633 ± 0.107	2.339 ± 0.099	2.428 ± 0.084	nd	nd	nd	nd	nd
C20:0	0.938 ± 0.032	0.963 ± 0.025	0.848 ± 0.047	nd	nd	nd	2.407 ± 0.103	2.391 ± 0.133	3.092 ± 0.205	1.892 ± 0.111	nd	nd	nd	nd	nd	nd
C20:1	1.853 ± 0.027	2.460 ± 0.100	nd	nd	nd	nd	nd	nd	nd	nd	nd	nd	nd	6.992 ± 0.277	nd	nd
C20:2	0.792 ± 0.050	0.844 ± 0.011	nd	nd	nd	nd	nd	nd	nd	nd	nd	nd	nd	0.972 ± 0.044	nd	nd
C20:3(α)	1.909 ± 0.113	0.783 ± 0.047	nd	nd	nd	nd	nd	nd	nd	nd	nd	nd	nd	nd	nd	nd
C20:3(γ)	0.657 ± 0.025	2.087 ± 0.231	1.271 ± 0.042	0.919 ± 0.022	1.012 ± 0.037	0.958 ± 0.011	nd	0.958 ± 0.021	1.728 ± 0.077	1.506 ± 0.054	1.426 ± 0.104	1.302 ± 0.088	1.381 ± 0.074	0.702 ± 0.011	nd	1.857 ± 0.021
C20:4	nd	nd	nd	nd	nd	nd	nd	nd	nd	nd	nd	nd	nd	nd	12.62 ± 2.547	nd
C20:5	nd	nd	nd	nd	nd	nd	nd	nd	nd	2.517 ± 0.231	nd	1.329 ± 0.070	nd	nd	nd	nd
C21:0	3.300 ± 0.745	nd	nd	nd	nd	nd	nd	nd	0.892 ± 0.022	nd	nd	nd	nd	nd	nd	nd
C22:0	nd	nd	4.621 ± 0.302	1.943 ± 0.057	2.198 ± 0.044	2.338 ± 0.066	nd	nd	nd	nd	nd	nd	nd	nd	nd	nd
C22:1	0.796 ± 0.031	nd	nd	nd	nd	nd	nd	nd	nd	nd	0.885 ± 0.019	nd	nd	nd	nd	nd
C22:2	4.714 ± 0.231	12.48 ± 1.558	nd	4.742 ± 0.241	nd	nd	nd	nd	nd	0.667 ± 0.022	nd	0.718 ± 0.044	nd	0.847 ± 0.038	nd	nd
C22:6	nd	0.744 ± 0.012	nd	nd	nd	nd	nd	nd	nd	0.767 ± 0.033	0.774 ± 0.054	0.768 ± 0.074	nd	nd	nd	nd
C23:0	1.453 ± 0.033	1.269 ± 0.230	0.954 ± 0.012	0.843 ± 0.021	0.932 ± 0.044	1.001 ± 0.078	nd	0.822 ± 0.066	1.048 ± 0.111	1.034 ± 0.123	1.047 ± 0.097	0.970 ± 0.022	0.937 ± 0.017	2.873 ± 0.544	nd	nd
C24:0	0.129 ± 0.011	0.132 ± 0.004	nd	0.243 ± 0.065	0.237 ± 0.077	0.378 ± 0.026	nd	0.342 ± 0.028	nd	0.452 ± 0.047	0.858 ± 0.088	0.891 ± 0.052	0.571 ± 0.014	0.241 ± 0.009	nd	nd
C24:1	0.692 ± 0.033	3.880 ± 0.455	nd	nd	nd	nd	nd	nd	nd	nd	nd	nd	nd	0.834 ± 0.123	nd	nd
ΣSFA	7.516 ± 0.333^a^	11.11 ± 1.154^b^	86.21 ± 4.858^k^	55.08 ± 2.455^d^	64.71 ± 5.177^f^	77.27 ± 6.255^j^	68.09 ± 4.748^g^	48.02 ± 2.110^c^	98.26 ± 8.478^l^	77.68 ± 7.188^j^	70.24 ± 5.125^h^	58.48 ± 2.131^e^	55.90 ± 1.999^d^	72.71 ± 4.422^i^	49.32 ± 2.155^c^	63.18 ± 3.331^f^
ΣMUFA	7.353 ± 0.015^j^	11.39 ± 0.811^m^	4.753 ± 0.721^g^	3.122 ± 0.434^de^	2.927 ± 0.158^cd^	2.775 ± 0.088^c^	3.675 ± 0.166^f^	4.842 ± 0.814^g^	0.463 ± 0.444^a^	8.892 ± 0.125^k^	6.712 ± 0.747^i^	5.370 ± 0.223^h^	3.456 ± 0.305^ef^	9.967 ± 1.000^l^	2.704 ± 0.357^c^	1.323 ± 0.059^b^
ΣPUFA	61.66 ± 3.585^k^	53.88 ± 2.222^j^	1.271 ± 0.123^ab^	25.02 ± 6.878^i^	22.34 ± 4.125^h^	0.958 ± 0.085^a^	2.300 ± 0.545^cd^	2.851 ± 0.121^d^	4.361 ± 0.333^e^	7.795 ± 0.158^f^	4.628 ± 0.545^e^	4.116 ± 0.133^e^	1.381 ± 0.325^ab^	20.23 ± 2.555^g^	25.02 ± 4.123^i^	1.857 ± 0.058^bc^
Σn-3	27.41 ± 1.775	0.744 ± 0.045	nd	1.262 ± 0.033	1.321 ± 0.021	0.958 ± 0.044	2.300 ± 0.066	1.893 ± 0.033	2.633 ± 0.055	5.623 ± 0.074	3.202 ± 0.244	2.097 ± 0.057	nd	nd	nd	nd
Σn-6	27.63 ± 2.112	39.88 ± 3.540	1.271 ± 0.058	19.01 ± 2.470	21.02 ± 3.011	nd	nd	0.958 ± 0.055	1.728 ± 0.152	1.506 ± 0.325	1.426 ± 0.111	1.302 ± 0.321	1.381 ± 0.122	19.38 ± 3.325	12.40 ± 2.111	1.857 ± 0.055
n-6/n3	1.008	53.60	nd	15.06	15.91	nd	nd	0.506	0.656	0.268	0.445	0.621	nd	nd	nd	nd
Σn-9	6.339 ± 0.411	10.13 ± 1.221	nd	nd	nd	nd	nd	0.578 ± 0.032	nd	1.390 ± 0.187	0.885 ± 0.055	nd	nd	7.826 ± 0.122	nd	nd
Total	76.53 ± 6.771^f^	76.38 ± 8.321^f^	92.23 ± 5.447^j^	83.22 ± 3.321^h^	89.98 ± 8.112^i^	81.01 ± 4.787^g^	74.06 ± 7.754^e^	55.71 ± 2.657^a^	103.1 ± 9.525^l^	94.37 ± 5.215^k^	81.58 ± 4.363^g^	67.96 ± 2.212^d^	60.73 ± 7.008^b^	102.9 ± 8.884^l^	77.04 ± 2.125^f^	66.36 ± 3.121^c^

a Results expressed as the mean ± standard deviation of triplicates. Different lowercase letters indicate significant differences among treatments based on Tukey’s test at *p* ≤ 0.05. Sample codes: A: 57 mL water and 0% digestate; B: 118 mL water and 0% digestate; C: 180 mL water and 0% digestate; D: 232 mL water and 0% digestate; E: 57 mL water and 10% digestate; F: 118 mL water and 10% digestate; G: 180 mL water and 10% digestate; H: 232 mL water and 10% digestate; I: 57 mL water and 20% digestate; J: 118 mL water and 20% digestate; K: 180 mL water and 20% digestate; L: 232 mL water and 20% digestate; M: 57 mL water and 30% digestate; N: 118 mL water and 30% digestate; O: 180 mL water and 30% digestate; P: 232 mL water and 30% digestate. SFA, MUFA, PUFA, and TFA stand for saturated, monounsaturated, polyunsaturated, and total fatty acids, respectively. n-3, n-6, and n-9 refer to omega-3, omega-6, and omega-9 fatty acids.

Monounsaturated fatty acids (MUFAs) ranged from 1.32% in sample P (232 mL of watering rate +30% digestate) to 11.39% in sample B. MUFAs are generally associated with beneficial cardiovascular effects, including improved lipid profiles and reduced inflammation. The higher MUFA levels in samples B, J, and N suggested that a watering rate of 118 mL for pot marigold cultivation, under the described conditions for experiments 1 and 2, may enhance MUFA biosynthesis, contributing positively to the oil’s health properties.

Polyunsaturated fatty acids (PUFAs) dominated the lipid profile, ranging from 0.95% in sample F to 61.66% in sample A. Higher PUFA contents were observed in samples A and B, with statistically significant differences from those with lower PUFA levels. These fats, including essential omega-3 and omega-6 fatty acids, are crucial for anti-inflammatory processes, cellular function, and cardiovascular health.^77,78^

Omega-3 fatty acids (ω-3) ranged from 0.74% in sample B to 27.41% in sample A. Omega-3s are renowned for their ability to reduce systemic inflammation, protect cardiovascular function, support neurological health, and lower triglycerides. Samples A and J, which exhibited the highest ω-3 levels, suggest that digestate treatments positively influence the enrichment of this crucial fatty acid group.

Omega-6 fatty acids (ω-6) were highest in sample B (39.88%) and sample A (27.63%) and lowest in sample H (0.95%). While omega-6s are essential for human health, excessive amounts relative to omega-3s can promote inflammatory responses. The reduction in ω-6 content with increasing digestate application, coupled with a concurrent rise in ω-3s, reflects a shift toward a healthier overall fatty acid balance, particularly evident in samples H to J.

An important additional aspect is the content of omega-9 fatty acids (Σ ω-9), which are a subset of MUFAs and include oleic acid. These ranged from 0.58% in sample H to 10.13% in sample B. Omega-9s are nonessential (since the human body can synthesize them), but they offer significant benefits, especially oleic acid, which has been linked to improved insulin sensitivity, reduced blood pressure, and better cardiovascular outcomes.^79^ Samples K and L, which exhibited the highest omega-9 levels, suggest that digestate fertilization positively influences the lipid profile.

Overall, the combination of low saturated fat, high polyunsaturated fat, elevated omega-3 and omega-9 contents, and moderate omega-6 levels makes pot marigold an excellent candidate for producing functional oils. Its lipid profile rivals that of many conventional vegetable oils and surpasses them in omega-3 concentration.^80,81^ This positions pot marigold oil as a natural, plant-based alternative with the potential to support cardiovascular health, reduce inflammation, and contribute to a balanced, heart-protective diet.

### 3.4 Environmental Metric Analysis

The EcoScale approach was used to evaluate the environmental sustainability of the carotenoid extraction method applied to pot marigolds in this study. For comparison, the results from two additional studies reporting alternative extraction techniques for this species were also analyzed. As shown in Table S5, the method used in this work achieved an EcoScale score of 89.8, the highest among the evaluated approaches. In contrast, the study by Yadav and Singh,^82^ despite achieving a slightly higher yield (0.7182 mg g^–1^), incurred significant environmental penalties (EcoScale score: 59) due to the use of a large volume and variety of organic solvents, including hexane, toluene, and ethanol, which not only increase the environmental burden but also pose safety risks. Similarly, the method reported by Piccaglia and collaborators^83^ received a lower score (58.1), primarily due to the use of chloroform, a hazardous solvent, and a higher solvent volume overall.

These findings are visually summarized in Figure 1. The method proposed in this study demonstrates minimal penalties, particularly in terms of safety and process duration. In contrast, the other methods show higher deductions, especially due to solvent hazards and extended extraction times. Notably, the current method required only 10 mL of acetone, was conducted at 25 °C, and completed in under 1 h, highlighting its operational simplicity and eco-efficiency. In summary, the acetone-based extraction used in this study demonstrated a favorable balance between yield and environmental performance, outperforming more complex and hazardous alternatives.

**Figure 1:**
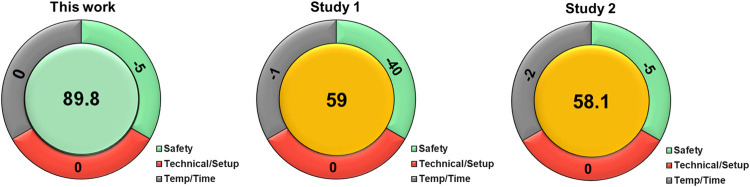
EcoScale score of total carotenoid content extraction from pot marigold (*C. officinalis* L.) plants.

### 3.5 Statistical Analysis and Multivariable Optimization

PCA was conducted to explore, from a multivariate perspective, the relationships among agronomic parameters, different irrigation regimes, and digestate concentrations, as well as their influence on plant growth, yield, and the nutritional and functional characteristics of marigold flowers. As illustrated in Figure 2, treatments are distinctly separated along two principal components (PC1 and PC2), which account for the majority of the variance in the data set. PC1 (40.5%) primarily reflects variables associated with vegetative growth and productivity, whereas PC2 (28.02%) corresponds to developmental timing including flowering and harvest stages.

**Figure 2:**
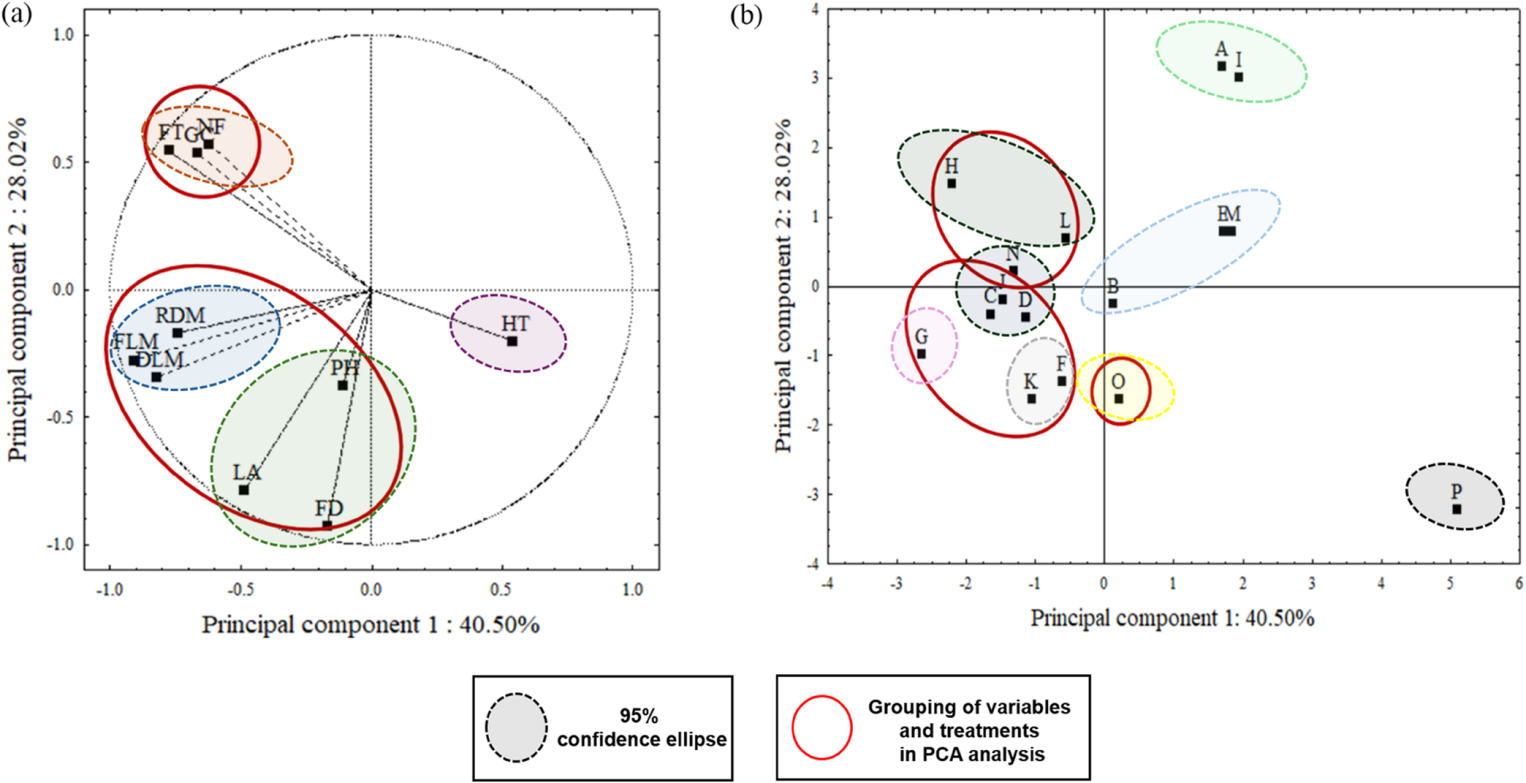
Principal component analysis of agronomic experimental data and the treatments of experimental design: (a) PCA loading plot and (b) PCA score plot. Legend: NF: Number of flowers; FD: Flower diameter; HT: Harvest time; FT: Flowering time; GC: Plant life cycle; PH: Plant height; LA: Leaf area; FLM: Fresh matter of the aerial part; DLM: Dry matter of the aerial part; RDM: Root dry matter. Sample codes: A: 57 mL water and 0% digestate; B: 118 mL water and 0% digestate; C: 180 mL water and 0% digestate; D: 232 mL water and 0% digestate; E: 57 mL water and 10% digestate; F: 118 mL water and 10% digestate; G: 180 mL water and 10% digestate; H: 232 mL water and 10% digestate; I: 57 mL water and 20% digestate; J: 118 mL water and 20% digestate; K: 180 mL water and 20% digestate; L: 232 mL water and 20% digestate; M: 57 mL water and 30% digestate; N: 118 mL water and 30% digestate; O: 180 mL water and 30% digestate; P: 232 mL water and 30% digestate.

Principal component 1 (PC1) mainly distinguished the agronomic variables associated with vegetative growth, including plant height (PH), leaf area (LA), fresh aerial biomass (FLM), dry aerial biomass (DLM), and root dry matter (RDM), which are predominantly located on the left side of the PCA plot.

In contrast, principal component 2 (PC2) differentiated the treatments based on phenological traits, including flowering time (FT), harvest time (HT), and plant life cycle (GC), which are in the upper left region of the plot. This indicates that plants in this area exhibited longer growth cycles and delayed development. Treatments F, G, J, and K (with 10% and 20% digestate and moderate irrigation volumes) are in the lower left quadrant, close to the PH, LA, FLM, DLM, and RDM vectors, suggesting a strong association with vegetative development, although it is not linked to early flowering. Treatment L (232 mL of water and 20% digestate) stands out in the upper left quadrant, near the vectors related to flowering and cycle duration, indicating that despite adequate irrigation and nutrition, the plants exhibited a longer cycle or delayed flowering. Treatments O and P (180 and 232 mL of water, respectively, with 30% digestate) are in the lower-right quadrant, distant from the main vectors, suggesting overall low agronomic performance or unbalanced responses, possibly due to the negative effects of nutrient excess. Treatments with 0% digestate are positioned in the upper right region, near a few relevant vectors, indicating low vegetative vigor and a tendency toward a longer cycle yet with poor overall efficiency.

Overall, PCA revealed that treatments F, G, J, and K, combining 10 and 20% digestate with moderate irrigation, were most strongly associated with structural plant development, while extreme treatments, both without digestate and with 30%, showed an inferior or unbalanced performance. The analysis highlighted the importance of applying intermediate digestate concentrations combined with adequate water volumes to maximize the vegetative growth without compromising the crop cycle. Therefore, regarding agronomic parameters, PCA proved to be a highly effective tool for identifying treatment groups with distinct agronomic characteristics, validating trends observed in the experimental data and supporting the selection of the most appropriate treatment based on productivity goals and plant development.

Simultaneously, a PCA was performed using the physical, chemical, and functional parameters, through the bioactive compounds, for all flower samples under different irrigation and digestate treatments, as shown in Figure 3.

**Figure 3:**
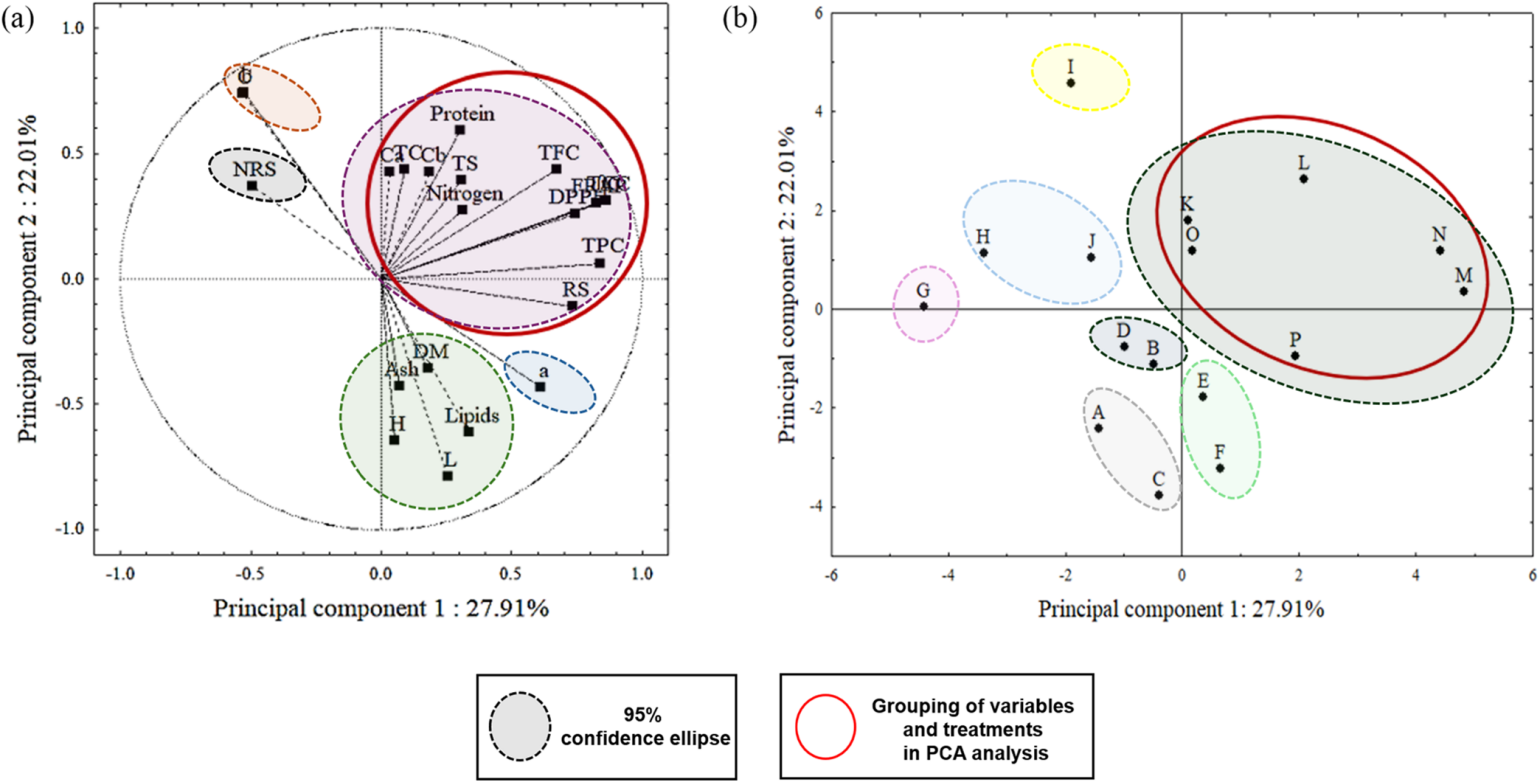
Principal component analysis for the physicochemical determinations of experiments with whole flowers and the experimental design treatments: (a) PCA loading plot and (b) PCA score plot. Legend: DM: Dry matter; RS: Reducing sugars; NRS: Nonreducing sugars; TS: Total sugars; Ca: Chlorophyll a; Cb: Chlorophyll b; TC: Total chlorophyll; TPC: Total phenolic compounds; TFC: Total flavonoids content; TCC: Total carotenoids content; β: β-Carotene; L: Lightness; a: Red-green value; b: Blue-yellow value; C: Chroma; H: Hue angle. Sample codes: A: 57 mL water and 0% digestate; B: 118 mL water and 0% digestate; C: 180 mL water and 0% digestate; D: 232 mL water and 0% digestate; E: 57 mL water and 10% digestate; F: 118 mL water and 10% digestate; G: 180 mL water and 10% digestate; H: 232 mL water and 10% digestate; I: 57 mL water and 20% digestate; J: 118 mL water and 20% digestate; K: 180 mL water and 20% digestate; L: 232 mL water and 20% digestate; M: 57 mL water and 30% digestate; N: 118 mL water and 30% digestate; O: 180 mL water and 30% digestate; P: 232 mL water and 30% digestate.

Upon examining Figure 3, PC1 (27.91%) was primarily associated with bioactive compounds, particularly pigments such as chlorophylls a and b, total chlorophyll, and total carotenoids, along with color-related attributes (L, a*, b*, Chroma, and Hue angle) and antioxidant activity (DPPH, FRAP, and β-carotene). These variables played a central role in distinguishing the different treatments, despite being minor constituents, due to their high functional relevance. Treatments with 20 and 30% digestate, especially those located in the upper quadrants of the PCA plot, showed a stronger correlation with these variables, suggesting that higher digestate concentrations, when combined with moderate irrigation levels, tend to enhance pigment accumulation and antioxidant capacity in pot marigold flowers. Treatments such as J, K, L, M, N, and O stood out in this regard, clustering closely with vectors associated with bioactive characteristics, indicating a favorable physiological response.

In contrast, treatments without digestate (A–D) and the one with 30% digestate under excessive irrigation (P) did not show a consistent pattern and appeared on the opposite side of the data distribution, suggesting reduced contributions to functional and visual quality likely due to nutritional deficiencies or osmotic stress caused by excess input. A positive association was also observed between antioxidant indicators (DPPH, FRAP, and β-carotene) and the levels of TPC and TFC, underscoring the role of balanced organic nutrient supply in stimulating the production of secondary metabolites.

These findings highlight the potential of marigold flowers rich in bioactive compounds for a wide range of applications, including functional foods and nutraceuticals such as teas, beverages, supplements, and energy bars; natural colorants and flavorings; cosmetic and dermocosmetic products with antioxidant and antiaging properties; and even phytotherapeutic and pharmaceutical formulations.

Furthermore, their application in biodegradable antioxidant packaging shows promise for enhancing food preservation and adding value to the sustainable development of innovative products.

## 4 Conclusions

The application of grape pomace digestate as an organic fertilizer for pot marigold (*C. officinalis* L. ‘Pôr do Sol’) proved to be an effective and sustainable strategy to enhance plant growth and flower biochemical quality. The anaerobically digested grape pomace provided a balanced supply of macronutrients (N, 1.65 g 100 g^–1^; P, 0.25 g 100 g^–1^; K, 0.16 g 100 g^–1^), micronutrients (e.g., Fe, 0.58 g 100 g^–1^; Zn, 0.024 g 100 g^–1^; Cu, 7.75 mg 100 g^–1^; Mn, 3.28 mg 100 g^–1^), and volatile fatty acids (propionic acid, 0.48 g 100 g^–1^; isobutyric acid, 1.28 g 100 g^–1^; isovaleric acid, 3.96 g 100 g^–1^), with no detectable toxic elements (As, Pb, Hg, Se), thereby improving substrate fertility and supporting nutrient recycling.

Across two seasonal greenhouse trials, moderate digestate concentrations (20–30%) under optimized irrigation regimes achieved the best agronomic outcomes. Excessive applications reduced biomass and flower number, likely due to nutrient imbalance or salinity stress, whereas moderate treatments significantly increased flowering, leaf area, and root development. Treatments combining 10–20% digestate with 118–180 mL water per day consistently produced vigorous growth and high-quality flowers.

Digestate fertilization also enhanced the biochemical composition, with higher concentrations of proteins, soluble sugars, carotenoids (notably β-carotene), phenolics, flavonoids, and antioxidant activity, key attributes for functional food, nutraceutical, and cosmetic applications. Seasonal effects were evident: summer favored greater sugar and carotenoid accumulation, while winter enhanced pigment and mineral retention, emphasizing the need for tailored nutrient–water management.

Importantly, the process achieved an EcoScale score of 89.8, substantially higher than comparable literature values, confirming its strong environmental performance and process greenness. Overall, grape pomace digestate represents a safe, nutrient-rich biofertilizer that advances circular bioeconomy principles, offering a scalable, low-input alternative to synthetic fertilizers for sustainable and multifunctional crop production systems.

## Supplementary Material



## Notes

Chemical composition of the soil substrate, physicochemical characteristics of the grape pomace digestate, color of whole flower extracts in both experiments, and EcoScale-assigned penalty points (PDF)
