# Supplementary material for: Sustainable Valorization of Grape Pomace Digestate as Fertilizer: Effects on the Agronomic and Biochemical Performance of Calendula officinalis L. under Variable Irrigation Regimes
Source: ACS Omega. 2026 Jun 19;11(25):36990–7011. doi: 10.1021/acsomega.6c00987 (PMC13325140; doi:10.1021/acsomega.6c00987)
Supplement: Supplementary file 1 [file ao6c00987_si_001.pdf]

SUPPLEMENTARY MATERIAL FOR:

Sustainable Valorization of Grape Pomace Digestate as Fertilizer: Effects on Agronomic and Biochemical Performance of *Calendula officinalis* L. under Variable Irrigation Regimes

Luiz Eduardo Nochi Castro[a,*], Renata Bachin Mazzini-Guedes[b], Maycon Diego Ribeiro[b], Larissa Resende Matheus[c], Daiane Leticia Quirino de Souza[d], Leomara Floriano Ribeiro[b], Leda Maria Saragiotto Colpini[b,c] , Tania Forster-Carneiro[a]

[a] School of Food Engineering (FEA), State University of Campinas (UNICAMP), Campinas, SP, Brazil

[b] Advanced Campus of Jandaia do Sul, Federal University of Parana (UFPR), Jandaia do Sul, PR, Brazil

[c] Graduate Program in Technology and Environmental Engineering, Palotina Sector, Federal University of Parana (UFPR), Palotina, PR, Brazil

[d] Institute of Chemistry (IQ), State University of Campinas (UNICAMP), Campinas, SP, Brazil.

*** Corresponding author:** l234211@dac.unicamp.br (Castro, L.E.N.)

Table S1. Chemical composition of the soil substrate

Parameters	Values	Unit
pH ($CaCl_2$)	5.7	—
P	97.55	mg dm^{-3}
K	1.31	cmolc dm^{-3}
S	55.84	mg dm^{-3}
Ca^{2+}	1.5	cmolc dm^{-3}
Mg^{2+}	3.58	cmolc dm^{-3}
Cu	1.45	mg dm^{-3}
Zn	3.73	mg dm^{-3}
Fe	65.37	mg dm^{-3}
Mn	4.45	mg dm^{-3}
B	0.4	mg dm^{-3}
Al^{3+}	0	cmolc dm^{-3}
H^+	2.39	cmolc dm^{-3}
Organic matter	6.53	%
Sum of bases	6.39	cmolc dm^{-3}
Cation exchange capacity	8.78	cmolc dm^{-3}
Base saturation	72.78	%

Table S2. Physicochemical characteristics of the grape pomace digestate

Parameters	Digestate	Unit
pH	8.520 ± 0.500	-
Electrical conductivity	0.240 ± 0.071	dS m^{-1}
Alkalinity	0.407 ± 0.020	g $CaCO_3$ 100 g^{-1}
Ammonia nitrogen	4.870 ± 0.310	mg $NH_3$100 g^{-1}
Total nitrogen	1.650 ± 0.210	g 100 g^{-1}
NO_3^-	< 0.010 ± 0.001	
NO_2^-	< 0.010 ± 0.001	
Total phosphorous	0.005 ± 0.001	
Total solids	5.670 ± 0.170	
Total fixed solids	1.820 ± 0.040	
Total volatile solids	3.850 ± 0.040	
Soluble protein	5.410 ± 0.380	
Total COD	4.690 ± 0.170	g O_2 g^{-1}
C/N	17.79 ± 2.127	-
Volatile fatty acids		
Propionic acid	0.480 ± 0.002	g 100 g^{-1}
Isobutyric acid	1.280 ± 0.060	
Isovaleric acid	3.960 ± 0.200	
Elemental composition		
Al	0.529 ± 0.012	g 100 g^{-1}
As	n.d.	
B	n.d.	
Ba	2.747 ± 0.254	10^{-3} g 100 g^{-1}
C	27.40 ± 3.580	g 100 g^{-1}
Ca	0.047 ± 0.001	
Cd	4.000 ± 0.152	10^{-3} g 100 g^{-1}
Cr	0.001 ± 0.000	g 100 g^{-1}
Cu	7.750 ± 0.236	10^{-3} g 100 g^{-1}
Fe	0.581 ± 0.003	g 100 g^{-1}
Hg	n.d.	
K	0.162 ± 0.001	
Mg	0.105 ± 0.004	
Mn	3.280 ± 0.022	10^{-3} g 100 g^{-1}
Mo	0.012 ± 0.001	g 100 g^{-1}
N	1.540 ± 0.032	

Na	1.610 ± 0.044	
Ni	0.380 ± 0.019	10^{-3} g 100 g^{-1}
P	0.250 ± 0.006	
Pb	n.d.	
S	0.531 ± 0.007	g 100 g^{-1}
Se	n.d.	
Zn	0.024 ± 0.001	

Table S3. Color of whole flowers extract experiment 1.

Sample	CIELab paramters					
	L*	a*	b*	C*	H°	Visual appearance
**A**	95.29 ± 0.51 ^{b}	-5.86 ± 0.03 ^{b}	30.14 ± 0.88 ^{ab}	30.70 ± 1.17 ^{a}	98.62 ± 0.79 ^{a}	
**B**	95.04 ± 0.61 ^{b}	-6.29 ± 0.11 ^{ab}	32.32 ± 0.28 ^{ab}	32.93 ± 0.29 ^{ab}	98.62 ± 0.54 ^{a}	
**C**	95.83 ± 0.69 ^{b}	-6.36 ± 0.14 ^{ab}	29.56 ± 0.25 ^{a}	30.24 ± 0.08 ^{a}	98.64 ± 0.94 ^{a}	
**D**	95.20 ± 3.41 ^{b}	-6.29 ± 0.03 ^{ab}	30.90 ± 0.48 ^{ab}	31.53 ± 0.89 ^{ab}	98.63 ± 0.19 ^{a}	
**E**	95.13 ± 3.03 ^{b}	-5.72 ± 0.20 ^{b}	29.91 ± 1.08 ^{a}	30.45 ± 1.03 ^{ab}	98.62 ± 2.65 ^{a}	
**F**	95.57 ± 1.50 ^{b}	-5.36 ± 0.16 ^{b}	26.89 ± 0.84 ^{a}	27.42 ± 0.81 ^{a}	98.63 ± 0.17 ^{a}	
**G**	93.54 ± 0.87 ^{ab}	-8.79 ± 0.03 ^{a}	47.69 ± 0.95 ^{ab}	48.49 ± 0.06 ^{ab}	98.61 ± 0.44 ^{a}	
**H**	94.42 ± 1.26 ^{ab}	-7.31 ± 0.15	37.74 ± 0.22 ^{ab}	38.44 ± 1.23 ^{ab}	98.62 ± 0.76 ^{a}	
**I**	92.36 ± 2.78 ^{a}	-8.59 ± 0.11 ^{a}	51.20 ± 0.41 ^{b}	51.92 ± 0.46 ^{b}	98.60 ± 3.69 ^{a}	
**J**	92.28 ± 0.23 ^{a}	-5.69 ± 0.24 ^{b}	39.90 ± 0.78 ^{ab}	40.30 ± 0.87 ^{ab}	98.57 ± 4.44 ^{a}	
**K**	93.34 ± 1.71 ^{ab}	-6.17 ± 0.04 ^{ab}	36.65 ± 2.39 ^{ab}	37.17 ± 0.48 ^{ab}	98.60 ± 0.82 ^{a}	
**L**	93.82 ± 1.08 ^{ab}	-6.24 ± 0.29 ^{ab}	36.02 ± 1.47 ^{ab}	36.56 ± 0.40 ^{ab}	98.60 ± 2.41 ^{a}	
**M**	94.54 ± 3.27 ^{ab}	-5.87 ± 0.01 ^{b}	32.05 ± 0.25 ^{ab}	32.58 ± 0.33 ^{ab}	98.61 ± 3.32 ^{a}	
**N**	94.71 ± 3.71 ^{ab}	-5.83 ± 0.46 ^{b}	31.74 ± 0.47 ^{ab}	32.27 ± 0.71 ^{ab}	98.61 ± 1.23 ^{a}	
**O**	92.19 ± 7.53 ^{a}	-5.55 ± 0.40 ^{b}	38.98 ± 0.44 ^{ab}	39.37 ± 0.65 ^{ab}	98.57 ± 1.49 ^{a}	
**P**	95.75 ± 1.39 ^{b}	-6.08 ± 0.03 ^{ab}	29.00 ± 1.42 ^{ab}	29.63 ± 0.25 ^{a}	98.64 ± 3.37 ^{a}	

*Results expressed as the mean ± standard deviation of triplicates. Different lowercase letters indicate significant differences among treatments based on Tukey's test at $p \leq 0.05$. Sample codes: A – 57 mL water and 0% digestate, B – 118 mL water and 0% digestate, C – 180 mL water and 0% digestate, D – 232 mL water and 0% digestate, E – 57 mL water and 10% digestate, F – 118 mL water and 10% digestate, G – 180 mL water and 10% digestate, H – 232 mL water and 10% digestate, I – 57 mL water and 20% digestate, J – 118 mL water and 20% digestate, K – 180 mL water and 20% digestate, L – 232 mL water and 20% digestate, M – 57 mL water and 30% digestate, N – 118 mL water and 30% digestate, O – 180 mL water and 30% digestate, P – 232 mL water and 30% digestate.

Table S4. Color of whole flowers extract experiment 2.

Sample	CIELab parameters					
	**L***	**a***	**b***	**C***	**Hº**	**Visual appearance**
**A**	94.49 ± 0.51 ^{ab}	-5.17 ± 0.03 ^{ab}	27.85 ± 0.88 ^{ab}	28.33 ± 1.17 ^{ab}	98.61 ± 0.79 ^{a}	
**B**	95.06 ± 0.61 ^{ab}	-5.70 ± 0.11 ^{ab}	29.14 ± 0.28 ^{ab}	29.69 ± 0.29 ^{ab}	98.62 ± 0.54 ^{a}	
**C**	95.63 ± 0.69 ^{ab}	-5.67 ± 0.14 ^{ab}	27.71 ± 0.25 ^{ab}	28.28 ± 0.08 ^{ab}	98.63 ± 0.94 ^{a}	
**D**	95.55 ± 3.41 ^{ab}	-5.57 ± 0.03 ^{ab}	27.57 ± 0.48 ^{ab}	28.13 ± 0.89 ^{ab}	98.63 ± 0.19 ^{a}	
**E**	95.29 ± 3.03 ^{ab}	-4.79 ± 0.20 ^{b}	26.72 ± 1.08 ^{ab}	27.15 ± 1.03 ^{ab}	98.61 ± 2.65 ^{a}	
**F**	95.69 ± 0.87 ^{ab}	-5.07 ± 0.03 ^{ab}	26.20 ± 0.95 ^{ab}	26.69 ± 0.06 ^{ab}	98.62 ± 0.44 ^{a}	
**G**	94.01 ± 1.26 ^{ab}	-8.58 ± 0.15 ^{a}	37.74 ± 0.22 ^{ab}	38.44 ± 1.23 ^{b}	98.61 ± 0.76 ^{a}	
**H**	95.10 ± 2.78 ^{ab}	-7.71 ± 0.11 ^{a}	36.30 ± 0.41 ^{ab}	37.11 ± 0.46 ^{ab}	98.64 ± 3.69 ^{a}	
**I**	94.55 ± 0.23 ^{ab}	-7.96 ± 0.24 ^{a}	38.99 ± 0.78 ^{b}	39.79 ± 0.87 ^{b}	98.63 ± 4.44 ^{a}	
**J**	95.86 ± 1.71 ^{ab}	-5.82 ± 0.04 ^{ab}	30.04 ± 2.39 ^{ab}	30.60 ± 0.48 ^{ab}	98.62 ± 0.82 ^{a}	
**K**	96.57 ± 1.08 ^{b}	-4.90 ± 0.29 ^{b}	22.49 ± 1.47 ^{a}	23.02 ± 0.40 ^{a}	98.64 ± 2.41 ^{a}	
**L**	95.15 ± 3.27 ^{ab}	-5.43 ± 0.01 ^{ab}	29.13 ± 0.25 ^{ab}	29.63 ± 0.33 ^{ab}	98.61 ± 3.32 ^{a}	
**M**	94.83 ± 3.71 ^{ab}	-4.71 ± 0.46 ^{b}	26.93 ± 0.47 ^{ab}	27.34 ± 0.71 ^{ab}	98.60 ± 1.23 ^{a}	
**N**	94.71 ± 3.71 ^{ab}	-5.83 ± 0.46 ^{ab}	31.74 ± 0.47 ^{ab}	32.27 ± 0.71 ^{ab}	98.61 ± 1.23 ^{a}	
**O**	92.19 ± 7.53 ^{a}	-5.55 ± 0.40 ^{ab}	38.98 ± 0.44 ^{b}	39.37 ± 0.65 ^{b}	98.57 ± 1.49 ^{a}	
**P**	95.75 ± 1.39 ^{ab}	-6.08 ± 0.03 ^{ab}	29.00 ± 1.42 ^{ab}	29.63 ± 0.25 ^{ab}	98.64 ± 3.37 ^{a}	

*Results expressed as the mean ± standard deviation of triplicates. Different lowercase letters indicate significant differences among treatments based on Tukey's test at $p \leq 0.05$. Sample codes: A – 57 mL water and 0% digestate, B – 118 mL water and 0% digestate, C – 180 mL water and 0% digestate, D – 232 mL water and 0% digestate, E – 57 mL water and 10% digestate, F – 118 mL water and 10% digestate, G – 180 mL water and 10% digestate, H – 232 mL water and 10% digestate, I – 57 mL water and 20% digestate, J – 118 mL water and 20% digestate, K – 180 mL water and 20% digestate, L – 232 mL water and 20% digestate, M – 57 mL water and 30% digestate, N – 118 mL water and 30% digestate, O – 180 mL water and 30% digestate, P – 232 mL water and 30% digestate.

Table S5. Ecoscale-assigned penalty points for the environmental analysis of total carotenoid content extraction from pot marigold (*Calendula officinalis* L.) plants: A Comparative Study with Other Relevant Research on Eco-friendliness and Sustainability.

Carotenoid source	Yield (mg TCC g^{-1})	Relative yield (%)	Extraction method	Reagents	Technical setup	Price/ Availability	Safety	Temperature/ Time	Ecoscale	Reference
Pot Marigold (*Calendula officinalis*)	0.643	89.53	Solvent	Acetone (10 mL)	0 (Common Setup)	0	-5	0 (25 ºC, < 1h)	89.8	This work
Pot Marigold (*Tagetes erecta*)	0.7182	100	Solvent	Hexane (130 mL) Acetone (17 mL) Ethanol (6 mL) Toluene (7 mL) KOH (0.8 mL) Water (1.2 mL) Na_2SO_4 (6.2 mL)	0 (Common Setup)	0	-40	-1 (25 ºC, < 24h)	59	[82]
Pot Marigold (*Calendula officinalis*)	0.217	30.21	Solvent	Acetone (100 mL) Chloroform (0.2 mL)	0 (Common Setup)	0	-5	-2 (Heating < 1h)	58.1	[83]
